# Human Platelet Lysate-Derived Nanofibrils as Building Blocks to Produce Free-Standing Membranes for Cell Self-Aggregation

**DOI:** 10.1021/acsnano.4c02790

**Published:** 2024-06-04

**Authors:** Cátia F. Monteiro, Maria C. Gomes, Pankaj Bharmoria, Mara G. Freire, João A. P. Coutinho, Catarina A. Custódio, João F. Mano

**Affiliations:** CICECO − Aveiro Institute of Materials, Department of Chemistry, University of Aveiro, Campus Universitário de Santiago, Aveiro 3810-193, Portugal

**Keywords:** human platelet lysate, ionic liquid, amyloid-like fibrils, free-standing membrane, 3D microtissues, cell self-aggregation

## Abstract

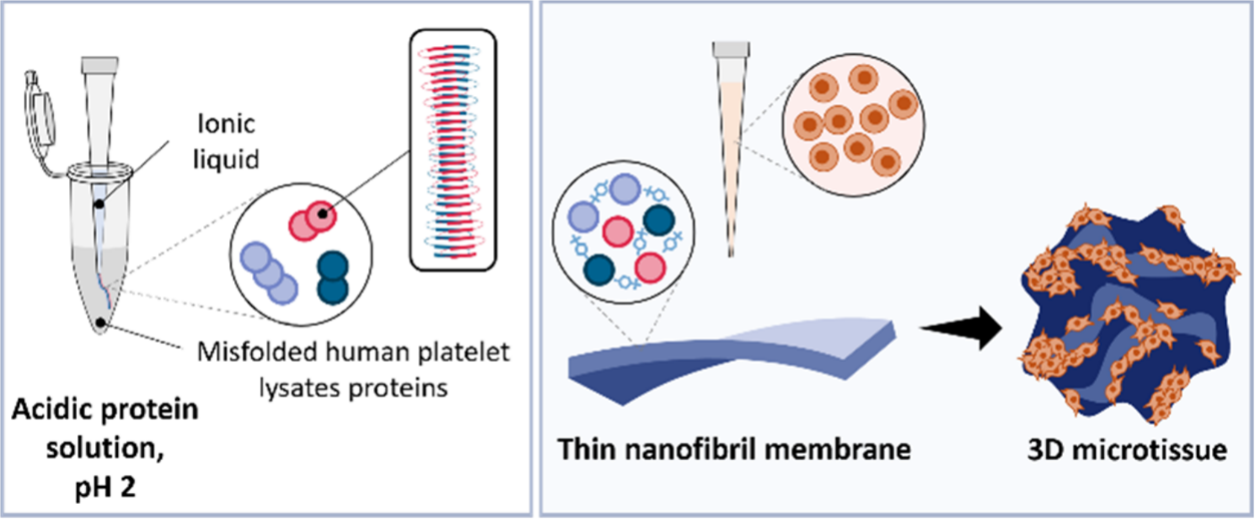

Amyloid-like fibrils are garnering keen interest in biotechnology as supramolecular nanofunctional units to be used as biomimetic platforms to control cell behavior. Recent insights into fibril functionality have highlighted their importance in tissue structure, mechanical properties, and improved cell adhesion, emphasizing the need for scalable and high-kinetics fibril synthesis. In this study, we present the instantaneous and bulk formation of amyloid-like nanofibrils from human platelet lysate (PL) using the ionic liquid cholinium tosylate as a fibrillating agent. The instant fibrillation of PL proteins upon supramolecular protein–ionic liquid interactions was confirmed from the protein conformational transition toward cross-β-sheet-rich structures. These nanofibrils were utilized as building blocks for the formation of thin and flexible free-standing membranes via solvent casting to support cell self-aggregation. These PL-derived fibril membranes reveal a nanotopographically rough surface and high stability over 14 days under cell culture conditions. The culture of mesenchymal stem cells or tumor cells on the top of the membrane demonstrated that cells are able to adhere and self-organize in a three-dimensional (3D) spheroid-like microtissue while tightly folding the fibril membrane. Results suggest that nanofibril membrane incorporation in cell aggregates can improve cell viability and metabolic activity, recreating native tissues’ organization. Altogether, these PL-derived nanofibril membranes are suitable bioactive platforms to generate 3D cell-guided microtissues, which can be explored as bottom-up strategies to faithfully emulate native tissues in a fully human microenvironment.

Amyloid fibrils emerged as protein structures correlated with the prevalence of multiple human degenerative health conditions, including Parkinson’s and Alzheimer’s disease, as a result of aberrant protein misfolding and consequent fibrillation.^1^ As insoluble protein aggregates with stable conformation, these amyloidogenic structures are rich in well-arranged and repetitive cross-β-sheet domains self-assembled in elongated fibrils.^2,3^ Despite the inherent toxicity attributed to pathological-related amyloid fibrils, a growing body of evidence has unveiled the occurrence of functional amyloid fibrils actively involved in a variety of beneficial biological processes within living systems.^4,5^ From the production of bacterial biofilms^6^ and melanosomes,^7^ to their crucial role in protein storage^8^ and cell signaling,^9^ functional amyloids are widespread in nature, emerging as attractive supramolecular materials for biomedical applications.^10^

Envisioning amyloid-like fibril application for cell culture purposes, a large number of animal-derived proteins have been explored to bioengineer fibril materials, including bovine whey protein β-lactoglobulin,^11,12^ bovine serum albumin,^13,14^ fibronectin,^15^ and hen egg lysozyme.^11,16,17^ Protein fibrils are usually fabricated by inducing the destabilization of the protein’s native conformation through environmental acidification and elevated temperatures. Seeking a thermodynamically favorable state, the misfolded proteins aggregate in highly organized cross-β-sheet-rich fibrillar structures stabilized by supramolecular interactions, such as hydrogen, hydrophobic, electrostatic, and van der Waals interactions.^18^ Nonetheless, if properly designed, ionic liquids (ILs) have emerged as biocompatible organic salts, exhibiting distinct effects on protein stabilization depending on protein features and the concentration and ionic components of the IL.^19^ While several studies have explored ILs as protein stabilizers,^20^ alternative approaches have demonstrated an interesting potential of these ionic compounds to act as promoters of protein fibrillation.^16,21,22^ For example, at low concentrations, ILs act as adjuvants and interact with proteins in associative forms through ion-specific interactions (e.g., triple ions, contact ion pairs) with the protein surface, where IL ions displace water from the hydration layer to destabilize proteins. At high concentrations, ILs act as cosolvents, where they form coclusters with the water in the hydration layer of proteins, stabilizing them via hydrophobic solvation, preferential exclusion, and hydrogen bonding.^19^ Contrary to stabilization/destabilization, protein fibrillation involves a conformational transition of protein/proteome to cross-β-type secondary structural conformation, which is driven by multiple interactions of the fibrillation agent (e.g., ionic liquid) with hydrophobic, hydrogen bonding, and ionic sites of the acid-denatured protein/proteome.^23,24^

Recent advances in amyloid-based materials have evidenced the potential of amyloid fibrils as cell-anchoring moieties to emulate cell–extracellular matrix (ECM) interactions, outperforming the cell adhesiveness observed with ECM protein components, such as collagen.^15,25^ The structural features of amyloid fibrils and their ability to interact with different molecules raised the opportunity to explore these nanomaterials as therapeutic vehicles of drugs and growth factors.^26^ In this line, protein fibrils have been investigated as functional building blocks to construct multiscale platforms in a bottom-up approach.^27^ Besides being used to produce fibril-based hydrogels,^28^ microcapsules,^29^ and membranes,^11^ these fibrillar nanostructures have been applied as bioactive reinforcements for hydrogels^12^ and as nanoscale structures for membrane functionalization.^30^ Despite the great advancements in nanostructured protein fibrils for biomedical applications, the exploration of alternative strategies remains crucial to recapitulate native tissues more realistically.

Tissues’ tridimensionality and ECM organization are fundamental features of the native microenvironment, offering a suitable physical support and biochemical environment to enable proper cell function. Spheroids are widely applied for tumor modeling, as their three-dimensional (3D) architecture, supported by the tight connections established between spheroid-forming cells, allows the generation of a necrotic core region surrounded by a proliferative zone. Although such oxygen and nutrient gradients are similar to those found in in vivo tumors, the lack of ECM-rich regions not only limits cell–ECM interactions but also hampers the faithful recreation of the tissues’ organization. Indeed, histological analysis of both healthy and tumor tissues has identified matrix-rich regions surrounding cell agglomerates.^31,32^ Concerning stem cell research, cell aggregates have been proposed as promising platforms to mimic stromal components in disease modeling and as therapeutic agents in clinical tissue injuries.^33,34^ However, the strong compaction of stem cells into spheroids promotes a necrotic core, a feature that hinders the controlled differentiation of the cells. Even though the incorporation of free microfibers,^35^ microparticles,^36^ and nanoscaffolds^37^ has been pursued to overcome the traditional spheroid limitations, these strategies still rely on forced-floating cell aggregation.

Aiming to offer a strategy for cell self-aggregation while incorporating a biomimetic matrix to reproduce tissues’ physiology (Figure 1), a simple and efficient method for producing thin and flexible membranes of human protein origin is proposed. The complex pool of proteins present in human platelet lysate (PL)^38^ was herein explored as a starting material to produce supramolecular functional protein fibrils in an instantaneous process induced by the IL cholinium tosylate. Exhibiting an amyloid-like structure, PL-based fibrils were used as building blocks to self-assemble in a nanopatterned free-standing membrane, which was hypothesized to provide cell-anchorage moieties, promoting cell–cell and cell–material interactions. The proposed cell-guided strategy can provide a more realistic in vivo*-*like tissue organization in a human microenvironment, emphasizing the importance of the cell–ECM interaction to bioengineer 3D cell platforms for biomedical applications.

**Figure 1:**
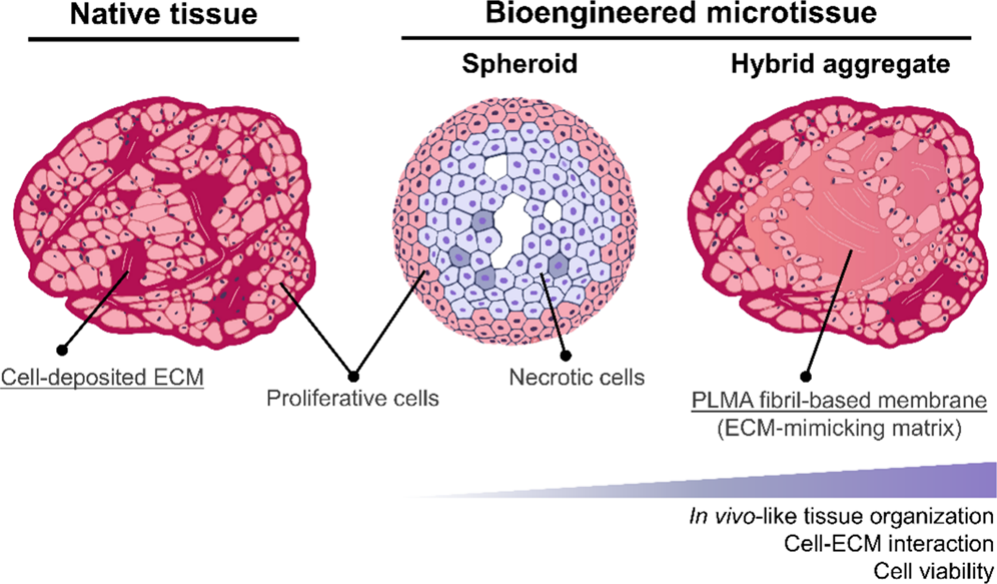
Concept of a bioengineered 3D microtissue to emulate the native tissue organization. Tissues are characterized by extracellular matrix (ECM)-rich regions surrounding proliferative cell agglomerates in a well-organized 3D structure that provides the suitable physical and biochemical microenvironment for proper cell function. Envisioning the recapitulation of fundamental features of native tissues, cell self-aggregated 3D structures generated from the cell-guided folding of a thin human methacryloyl platelet lysate (PLMA) fibril-based membrane are proposed as an alternative to the traditional spheroids. Besides reproducing the in vivo-like tissue organization, these bioengineered microtissues render enhanced cell–ECM interaction and cell viability.

## Results and Discussion

In native tissues, the ECM abundantly deposited by tissue-associated cells is responsible for regulating several cellular functions and affects tissue properties through its morphological organization and biomechanical features.^39^ Although great advances in proteinaceous biomimetic hydrogels have provided robust physiological and pathological models, amyloid-like fibrils are gaining momentum as protein-derived structures with huge potential to better emulate tissues’ structure and mechanical properties.^25,40,41^ So far, animal-derived proteins are the preferred materials to produce fibril-rich constructs. Nonetheless, the interest in human-derived biomaterials has steadily evolved given the reduced risk of immunogenicity and the absence of ethical concerns related to animal welfare.^42^ Recent studies have proposed a human methacryloyl platelet lysate (PLMA)-based hydrogel as a bioactive and functional material to support stem cell and tumor spheroid viability and invasion.^43−45^ In this line, PL- and PLMA-derived proteins were herein chosen as promising biomaterials to develop fibril-based 3D platforms for stem cell and tumor research.

### Ionic Liquid Induces Instantaneous Fibrillation of PL Proteins

**Figure 2:**
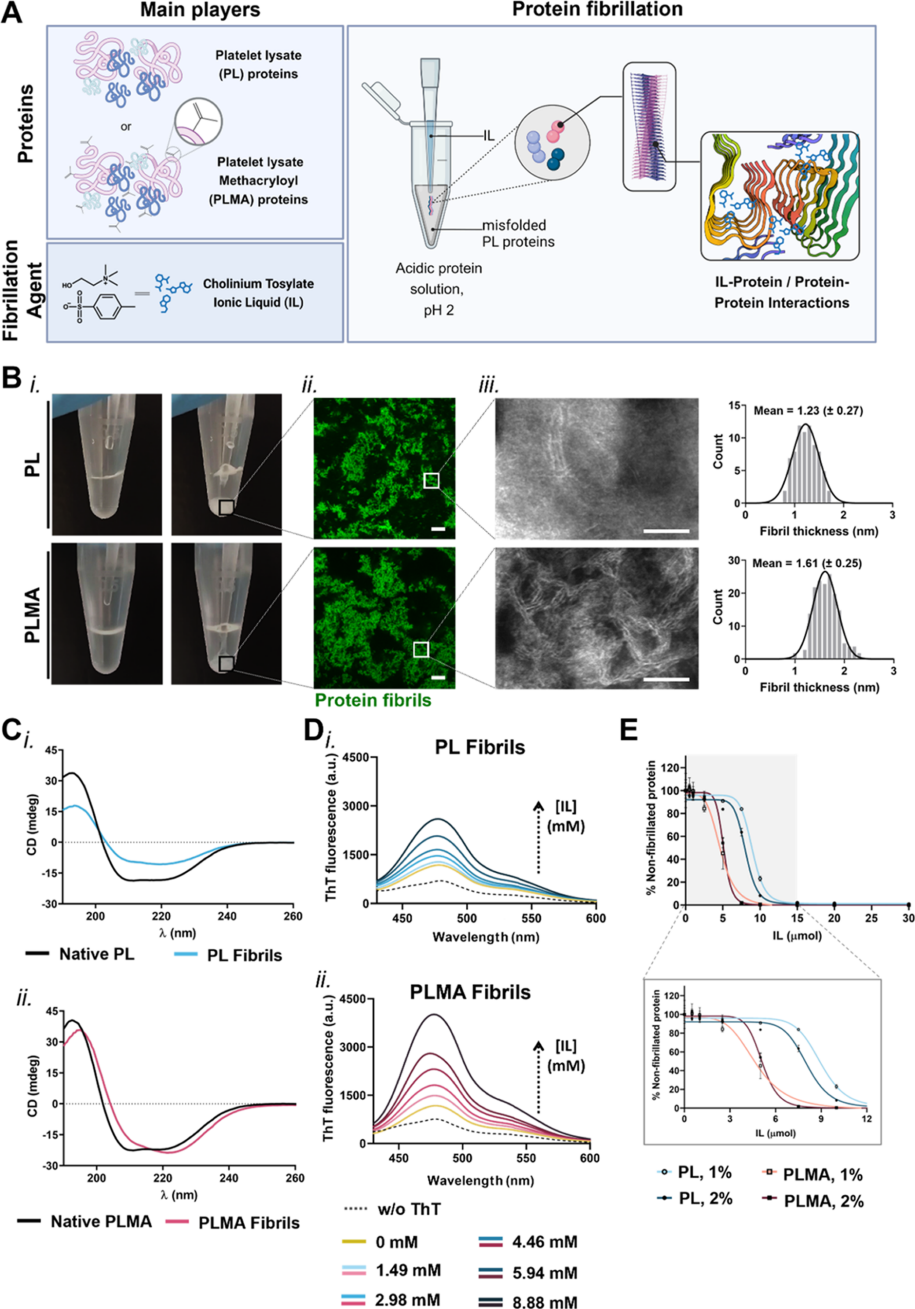
Production and characterization of human platelet lysate-derived amyloid-like fibrils. (A) Schematic illustration of the method used to produce protein fibrils from platelet lysate (PL) and methacryloyl platelet lysate (PLMA) as the main proteinaceous players. The addition of the fibrillation agent ionic liquid (IL) cholinium tosylate ([Cho][TOS]) to a denatured protein solution, induced by an acidic environment (pH 2), instantaneously prompts protein fibrillation. The produced amyloid-like fibrils rich in cross-β-sheet structures are mediated by IL–protein and protein–protein interactions. Created with BioRender.com. (B) (i) Time-lapse digital photographs demonstrating the instantaneous fibrillation of PL and PLMA acidic solutions upon IL addition, forming a macroscopically visible whitish protein precipitate (see Supporting Movies 1 and 2). (ii) Confocal laser microscopy and (iii) transmission electron microscopy (TEM) images of the protein fibrils, exhibiting a fibrillar structure. Scale bar: 10 μm and 50 nm. (iii) Protein fibril size distribution histogram measured from the TEM images. (C) Circular dichroism (CD) spectra of native (i) PL and (ii) PLMA protein solutions (solid black lines) and correspondent fibrils (solid color lines). (D) Fluorescence spectra of thioflavin T (ThT) labeled (i) PL and (ii) PLMA fibrils produced with increasing amounts of IL. (E) Efficiency of protein fibrillation by the quantification of non-fibrillated protein in the 1 and 2% (w/v) PL and PLMA solutions for different amounts of IL. Data are presented as mean ± standard deviation (SD) (*n* ≥ 3).

Protein denaturation was primarily triggered by an acidic environment (pH 2), disrupting the noncovalent interactions (hydrogen, hydrophobic, and electrostatic bonds) that play a major role in stabilizing the proteins’ native secondary conformation. Such structural unfolding exposes the hydrophobic amino acid side chains strategically arranged in the protein core, which are known to serve as nucleation sites for the formation of β-sheet-rich structures in protein fibrils.^3,46,47^ Afterward, the addition of the IL cholinium tosylate ([Cho][TOS]) instantaneously prompted the formation of a whitish precipitate, suggesting protein aggregation by interaction with the IL (Figure 2A,Bi and Supporting Movies 1 and 2). The structural characterization of those precipitates showed small protein-based units, exhibiting a fibrillar structure (Figure 2Bii,iii). Interestingly, the nanofibrils formed from the PLMA proteins presented a more well-defined and uniform fibril morphology, with higher thickness (1.61 (±0.25) nm) compared to PL ones (1.23 (±0.27) nm), suggesting a distinct protein–IL interaction between both proteins’ samples (Figure 2Biii). This protein fibril size, of only a few nanometers in width, aligns with previous reports using β-amyloid peptides and fibrils extracted from tissues.^16,48−50^ The instantaneous fibrillation of both PL/PLMA proteins contrasts with the amyloid fibril formation mechanism characterized by a lag phase of oligomer formation, followed by an exponential phase, where the nucleated fibril growth mechanism occurs (Figure S1A).^51^

To evaluate whether these fibril-like nanounits resulted from a conformation transition toward β-sheet-rich domains, the protein secondary structural changes were analyzed using circular dichroism (CD) and Fourier transform infrared (FTIR). The CD spectra analysis revealed that, while minimal changes were observed for the fibrillated PL, a conformational transition from α-helix to cross-β-sheet was evidenced for PLMA upon fibrillation with IL (Figure 2C). Such PLMA structural transition is characterized by the red shift of the positive band from 193 to 195 nm and the singular negative band at 221 nm. In FTIR, the deconvolution of the amide I region (1600–1700 cm^–1^) in the spectra revealed an increase in the peak area correspondent to β-sheet and β-turn structures in the PL and PLMA fibrils, compared to α-helix (Figure S1B–D). Moreover, specific peaks identified in the IL spectrum were also detected in the PL/PLMA fibril spectra, even after a fibril washing step to remove IL excess (Figure S1B). These conformational changes confirmed that [Cho][TOS] was an active intermediator of the instantaneous fibrillation phenomenon characterized by β-sheet-rich structure formation in PL-derived proteins, with a more prominent effect in PLMA. Indeed, ILs have been explored as modulators of intra- and intermolecular interactions, mediating the formation of highly ordered amyloid-like fibrils by noncovalently interacting with amino acids.^16,22,52^ In particular, [Cho][TOS] was previously reported to induce the formation of long and highly branched fibrils from chicken egg white proteome,^22^ however morphologically different from the fibrils herein described. This difference in morphology can be attributed to the distinct and complex composition of the PL proteins,^38^ comprising proteins including serum albumin, serotransferrin, a diversity of immunoglobulins, complement factors, and others (Table S1). The fibrils resulting from this pool of proteins highlight the importance of the material nature and components to trigger the formation of singular structures. Hydrophobic amino acids are recognized as the main mediators of β-sheet-rich structures assembling during protein fibrillation processes.^46,47^

In an attempt to correlate molecular composition with a fibril structure, a thorough analysis of the hydrophobic amino acid and secondary structure content in the native conformation was performed for the most abundant PL proteins (Table S1). Despite the hydrophobic amino acid content being similar among the analyzed proteins, most of them revealed a higher number of β-strand domains and an increased percentage of amino acid participating in their secondary structures. Although proteins are denatured before fibrillation, the high β-strand domain content is hypothesized to contribute to the instantaneous and complete PL fibrillation, resulting in the herein reported small fibrillar structures.

Previous molecular docking studies demonstrated that the protein–IL interaction is mainly mediated by the tosylate anion ([TOS]^−^), which favorably establishes hydrogen bonds and hydrophobic interactions with the amino acid side chains.^22^ The positively charged amino acids, arginine, histidine, and lysine, and the hydrophobic amino acids, alanine, valine, tyrosine, and phenylalanine, are the most involved in these interactions. Considering these [TOS]^−^ interaction preferences, the chemically modified PLMA was hypothesized to be more prone to [Cho][Tos]-induced fibrillation due to the hydrophobic nature of the methacryloyl groups. In fact, about 85% of the free amines in the PL was functionalized with methacrylic moieties, unveiling the high reactivity of methacrylic anhydride toward amine groups, as reported elsewhere (Figure S1E).^53^ To understand if the higher hydrophobicity of the PLMA proteins influenced β-sheet structure formation, changes in the thioflavin T (ThT) fluorescence were studied with increasing amounts of IL in the presence of the same amount of protein (Figure 2D). By specifically binding to β-sheet domains, the increase in the ThT fluorescence indicated an increase in β-sheet content in the PLMA samples, compared to the same amount of PL proteins.^54^ This is in accordance with previous studies correlating the increased hydrophobic content and its exposure to the higher propensity of proteins to generate amyloidogenic fibrils.^55^

In order to compare the fibril formation efficiency from PL and PLMA proteins and evaluate the influence of protein concentration on fibrillation, protein solutions at 1 and 2% (w/v) were prepared. The higher efficiency of PLMA proteins to form fibrils was further confirmed through the quantification of the non-fibrillated protein, revealing a faster consumption of proteins for both 1 and 2% (w/v) PLMA compared with the PL (Figure 2E). The same was inclusively evident through the macroscopic observation of the whitish precipitate formation (Figure S2A). Moreover, the results demonstrated a fibril formation dependency on protein and IL amounts, with faster protein fibrillation for the more concentrated protein solution or for higher amounts of IL. Nonetheless, for IL quantities equal or superior to 15 μmol, the consumption of the protein content is quantitative, independent of the concentration of the initial protein solution (1 or 2% (w/v)). This demonstrates that more fibrils can be obtained simply by changing the concentration of the protein solution. Sodium dodecyl sulfate polyacrylamide gel electrophoresis (SDS-PAGE) further evidenced that the main proteins present in PL and PLMA were involved in the fibrillation process as a result of the total consumption of the protein in fibril formation (Figure S2B).

Overall, PL and PLMA fibril characterization results demonstrated the feasibility of IL [Cho][TOS] to instantaneously produce supramolecular functional protein fibrils through a simple and cost-effective method. However, considering the increased fibril formation efficiency of PLMA and the enhanced robustness of its fibrillar morphology, we chose PLMA fibrils to develop human protein-derived free-standing membranes to explore an efficient and reproducible strategy for cell self-aggregation.

### Ultrathin Free-Standing Micromembranes with Controlled Geometry Are Formed Using PLMA

**Figure 3:**
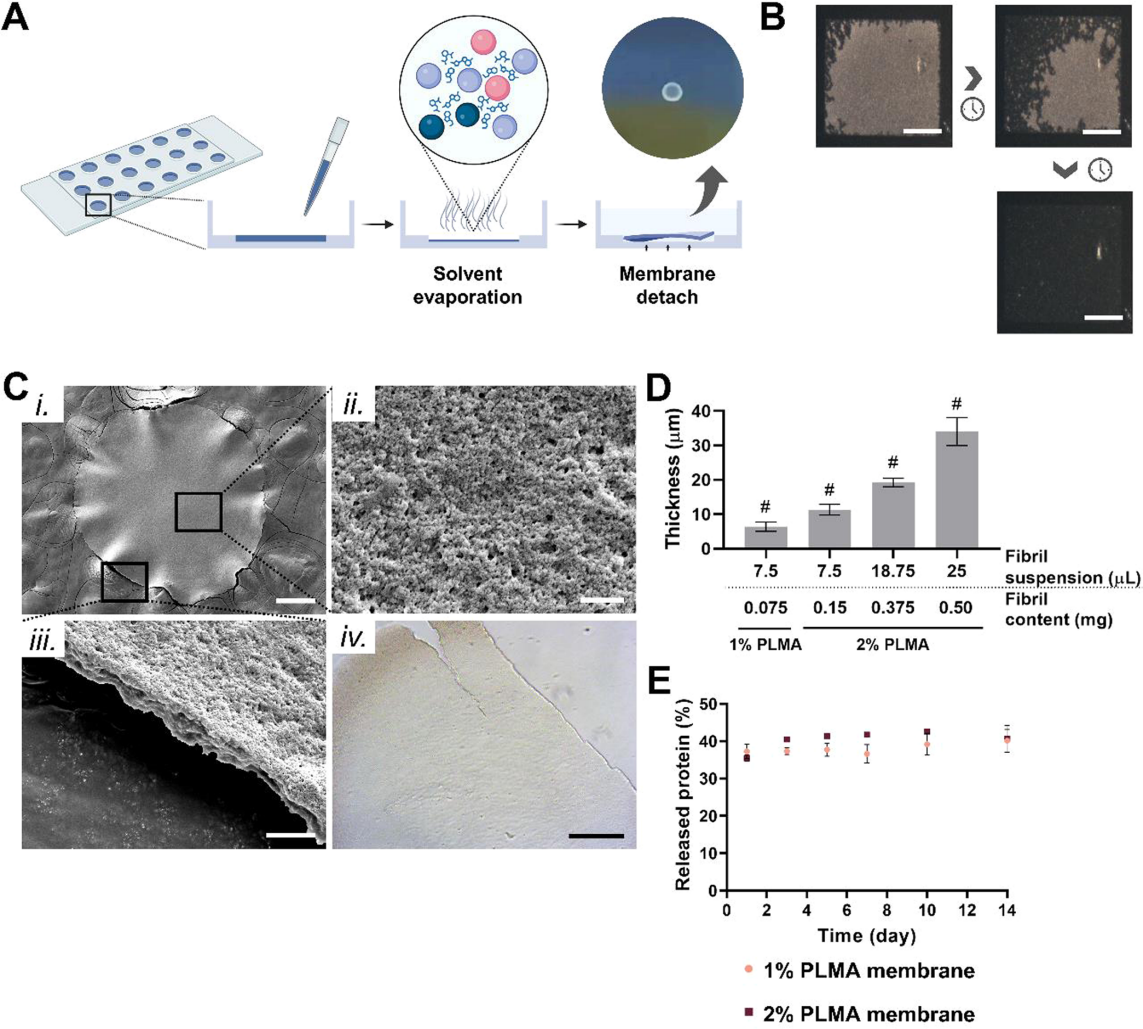
Fabrication and structural characterization of PLMA-based free-standing membranes. (A) Schematics of the approach used to fabricate the protein fibril-derived free-standing membranes. The membrane formed by solvent evaporation and mediated by fibril–fibril and fibril–IL interactions detaches from the surface after rehydration, revealing a semitransparent aspect: see Supporting Movie 3 to visualize membrane detachment from the surface and membrane flexibility. Created with BioRender.com. (B) Optical micrographs of the fibril unit assembly into a membrane by solvent casting. Scale bar: 500 μm. (C) Scanning electron microscopy (SEM) images of (i) the overall PLMA fibril membrane (scale bar: 200 μm) and close-up demonstration of the (ii) nanoscale topography of the membrane surface (scale bar: 2 μm) and (iii) membrane thickness (scale bar: 5 μm). (iv) Differential interference contrast image of the PLMA fibril-derived membrane showing a fractured region as a representation of the membrane brittleness to localized forces. Scale bar: 200 μm. (D) Thickness of PLMA fibril membranes prepared with distinct protein content from different volumes of 1 and 2% PLMA solution. (E) Protein release quantification of 1 and 2% (w/v) PLMA fibril-derived membranes incubated in cell culture conditions for 14 days. Data are presented as mean ± SD (*n* ≥ 3).

Harnessing protein fibrils as functional building blocks to produce free-standing membranes remain underexplored and limited to drug delivery,^30^ food industry,^56^ and water filtration^57^ applications. Additionally, the incorporation of plasticizers^11^ and other natural or synthetic polymers^57^ seems to be crucial for membrane/film formation. Herein, the fabrication of free-standing membranes based on PLMA protein fibrils of human origin is proposed as a great nanostructured multifunctional biomaterial for biomedical bottom-up approaches. To produce these free-standing membranes, the PLMA fibril suspension was cast on a plastic surface (ibidi μ-slide angiogenesis plate), and as water molecules evaporated, protein fibrils aggregated in a well-arranged manner creating an ultrathin membrane (Figure 3A). When rehydrated, this thin semitransparent membrane easily detached from the surface, maintaining its structure and unveiling an interesting intrinsic flexibility. To observe the fibril drying process more clearly, the protein fibril suspension was cast on a superhydrophobic–superhydrophilic microarray, formed on a glass slide, where the superhydrophobic regions confine the solution in the wettable spots. This membrane formation approach revealed a radial evaporation process progressing from the thinner to the thicker region of the droplet (Figure 3B and Supporting Movie 3). Such patterned surfaces can also provide a straightforward and scalable method of producing free-standing fibril membranes with a controllable shape and size. The highly stable fibril–fibril interaction established during membrane formation is hypothesized to be mediated by excess IL presented in the fibril solution, creating IL–fibril interactions. Indeed, several studies have been reporting that amyloid fibril aggregation is supported by amyloid interaction with diverse ECM components, such as glycosaminoglycans.^58^ Besides that, amino acid residues located in the outer region of PLMA fibrils can establish non-IL-mediated hydrogen, hydrophobic, and electrostatic interactions.^59,60^ Surface analysis of the fabricated free-standing membranes by scanning electron microscopy (SEM) revealed a topographical roughness and a membrane thickness in a few micrometer range (Figure 3C,i–iii). Such surface nanotopography conferred by the aggregation of fibril units is expected to enhance cell adhesiveness and spreading.^61^ This simple approach of integrating well-defined nanoscale topography has the potential to provide more biomimetic nanopatterns to control cell fate and morphology.^62^ The PLMA fibril membrane is also characterized by its brittleness, a feature explained by the weak supramolecular nature of the fibril–fibril interactions, coupled with the ultrathin thickness of the membrane (Figure 3C,iv). In addition, the thickness of the PLMA fibril membranes can be modulated by producing the membrane with different amounts of PLMA fibrils (Figures 3D and S3A). As expected, a higher fibril content renders thicker free-standing membranes, which are hypothesized to result in membranes with higher stiffness. Indeed, previous studies have reported that an increase in film/membrane thickness results in higher bending stiffness.^63,64^ Of note, 0.05 mg of fibrils (corresponding to 5 μL of 1% PLMA fibril suspension) is the lower amount of fibrils that render a robust and stable membrane.

To address the feasibility of these free-standing PLMA membranes for cell culture purposes, their integrity was evaluated by incubating them in cell culture medium under standard cell culture conditions. For that, PLMA membranes were fabricated from 1 and 2% (w/v) protein solutions, leading to a 2-fold increase in fibril density within the 2% PLMA membrane in comparison to the 1% counterpart. The quantification of the released protein revealed that 35–40% of the membrane protein content was released during the first 24 h after membrane rehydration (Figure 3E). It was hypothesized that the medium composition (e.g., salts, proteins, and amino acids, particularly the hydrophobic ones) could destabilize the noncovalent interactions established between IL ions and the amino acid side chains. However, the protein release during the following days was residual, even with a change in cell culture medium, demonstrating the highly stable network established between protein fibrils. Actually, PLMA fibril membranes maintained their macroscopic integrity when incubated in cell culture conditions for 14 days (Figure S3B). As such, the high amount of protein released at the beginning of the experiment is certainly protein fibrils not integrated into the membrane and released during the rehydration step. This phenomenon can be related to the limitation of IL available to fully intermediate fibril–fibril interactions in the membrane.

Knowing that protein fibrils are released from the PLMA membrane, concerns related to fibril cytotoxicity arose owing to amyloid fibril involvement in several human diseases.^1^ Besides the well-known detrimental role of amyloid plaques on cells,^65^ recent advances in amyloid toxicity mechanisms have suggested amyloid oligomers as one of the main responsible factors for cell membrane damage and consequent cell death^66^ in amyloidogenic diseases. However, a growing body of evidence has demonstrated that functional amyloid-like fibril structures can be bioengineered from natural or synthetic polymers and used as scaffolds for 3D cell culture, without disrupting membrane homeostasis and inducing cell death.^67^ Indeed, the ECM-resembling biomimetic topography conferred by amyloid-like fibrils was found to improve cell adhesion and proliferation^68^ and control cell differentiation.^28^ Therefore, to determine the cytotoxicity potential of the released fibrils, increasing amounts of free fibrils fabricated from 1 or 2% (w/v) PLMA solution were added to two-dimensional (2D) monolayers of bone marrow-derived mesenchymal stromal cells (hBM-MSCs) and osteosarcoma MG-63 tumor cell line and cultured for 3 days. Of note, the range of PLMA fibril quantity used in this experiment includes the amount of fibrils used to form the membrane. Cell viability quantification revealed that neither PLMA protein fibrils nor IL alone exhibited cytotoxic effects (Figure S4A,B). The cytocompatibility of the protein fibrils can be associated with the complete fibrillation mechanism instantaneously obtained after IL addition, hindering the existence of a fibril formation lag phase, during which fibril oligomers have detrimental effects on cell viability.^69^ Additionally, the presence of the IL intertwined in these fibrillar structures (as confirmed by FTIR) can hamper their interaction with cell lipid membranes, as previously reported, for glycosaminoglycans and other molecules that modulate fibrillation.^70^ Moreover, F-actin microfilament alignment was not affected by culturing the cells in the presence of PLMA fibrils (Figure S4C), demonstrating that both dispersed fibrils and fibril-based membranes are suitable supramolecular functional materials for cell scaffolding purposes.

### Cells Can Self-Aggregate with Free-Standing PLMA Membranes to Form Biomimetic Microtissues

**Figure 4:**
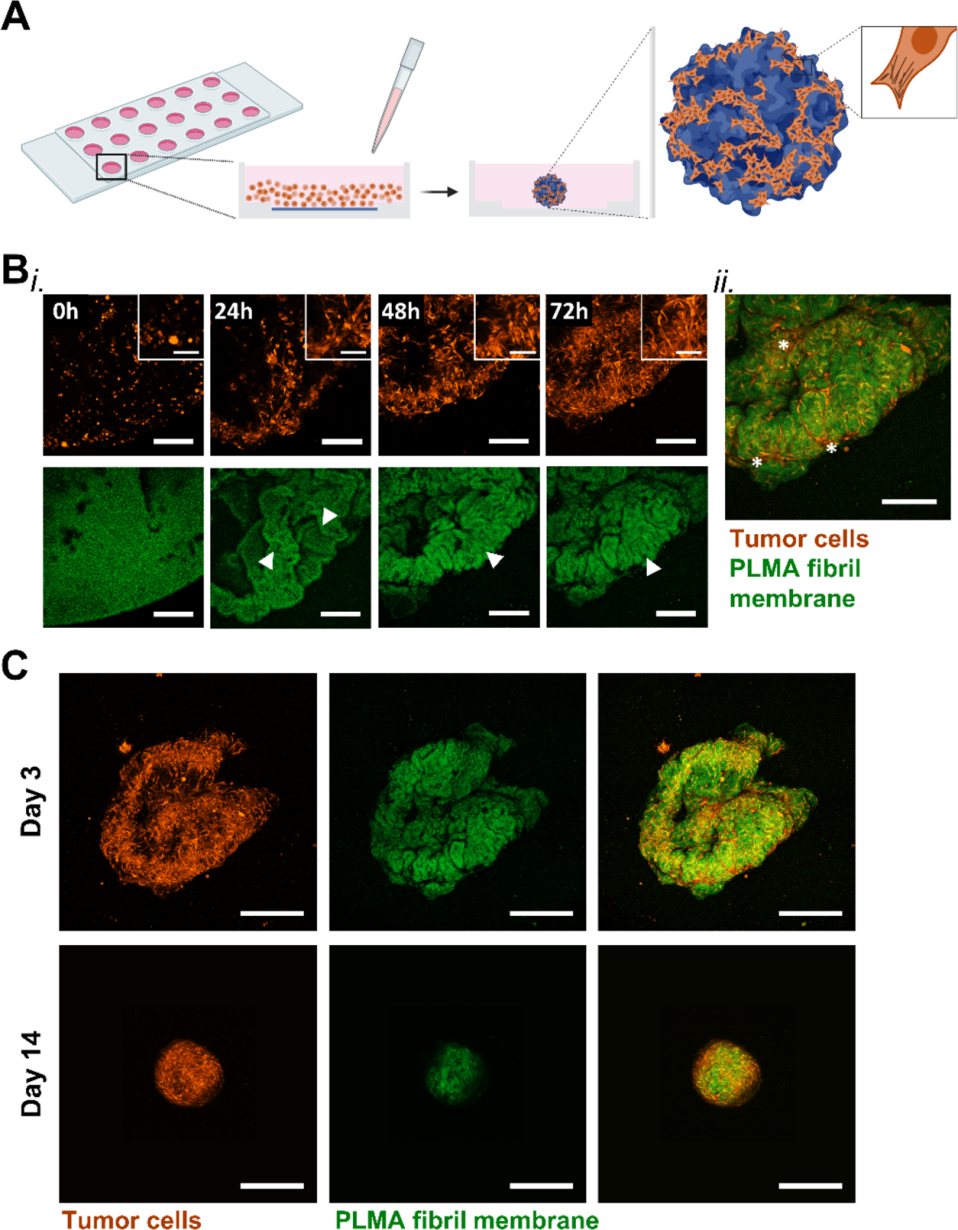
Cell–membrane interaction as a driving force for cell self-aggregation formation. (A) Schematic illustration of the PLMA fibril-derived membrane and the traction forces exerted by cells seeded on the top of the membrane, driving the formation of membrane folds and ultimate complete folding. Created with BioRender.com. (B) (i) Images captured by confocal laser microscopy during live imaging experiment (Supporting Movie 5) over 3 days of cells/membrane aggregates in culture. Scale bar: 250 μm. Magnified views of those images are presented in the upper right corner. Scale bar: 100 μm. Arrowheads highlight the membrane folds forming during membrane folding. (ii) Confocal laser microscopy image of the merged cells and PLMA membrane showing cells stretched between the membrane folds (highlighted with *). (C) Confocal laser microscopy images of the overall cell/membrane aggregate formed after 3 and 14 days in culture. Scale bar: 500 μm.

Leveraging the biocompatibility of the protein fibrils and membrane nanotopography facilitated by fibril–IL–fibril supramolecular interactions, PLMA fibril-based membranes were explored as adhesion platforms to bioengineer healthy and tumor models in a fully human microenvironment (Figure 4A). For this purpose, previously used hBM-MSCs and MG-63 cell lines were employed to assess the cell adhesion to the membrane. Based on previous reports, these cell types successfully adhered and proliferated in PLMA hydrogels, either as invading cell spheroids^43^ or combined in a coculture 3D tumor model.^44,45^ The great composition and biochemical properties of this human source of proteins were also evidenced by producing self-assembly PL microparticles that facilitated hBM-MSC adhesion and self-aggregation.^38^ When seeded on the top of the fibril-derived membrane produced on a flat surface, both cell types exhibited adhesion within 4 h (Figure S5A). The 3-day live imaging experiment with red fluorescent protein (RFP)-expressing MG-63 cells further demonstrated the propensity of these tumor cells to adhere, showing cell protrusions at 24 h of culture (Figure 4B and Supporting Movie 5). This experiment also evidenced their high migratory ability as they effectively folded the membrane. Moreover, the formation of membrane folds was observed, between which cells were spreading and self-aggregating as a result of strong cellular contractile forces, ultimately fostering membrane folding from the periphery. Such cell behavior suggests that the presented system enabled the free mobility of cells and the establishment of cell–material and cell–cell communication crucial for tissues’ function. This cell-mediated membrane folding was previously reported as a process with great potential to render high-reproducible 3D cell sheets with controllable patterns,^71^ recapitulating the cell–ECM interactions intermediated by focal adhesions.^72^ Over the 14-day culture period, it was observed that the cell aggregate became densely packed, with the majority of cells arranged in the outer region of the aggregate, while the PLMA fibril membrane was packed within (Figures 4C and S5B). This cell-guided assembly into a 3D structure demonstrates that the high area/volume of the free-standing membranes is effective in mediating the formation of hybrid microtissues and could be an alternative to bulky particles (usually spherical).^73^ To the best of our knowledge, no previous studies have reported a fully human protein fibril-based supramolecular free-standing membrane serving as a functional platform for cellular self-organization in in vivo-like 3D structures.

**Figure 5:**
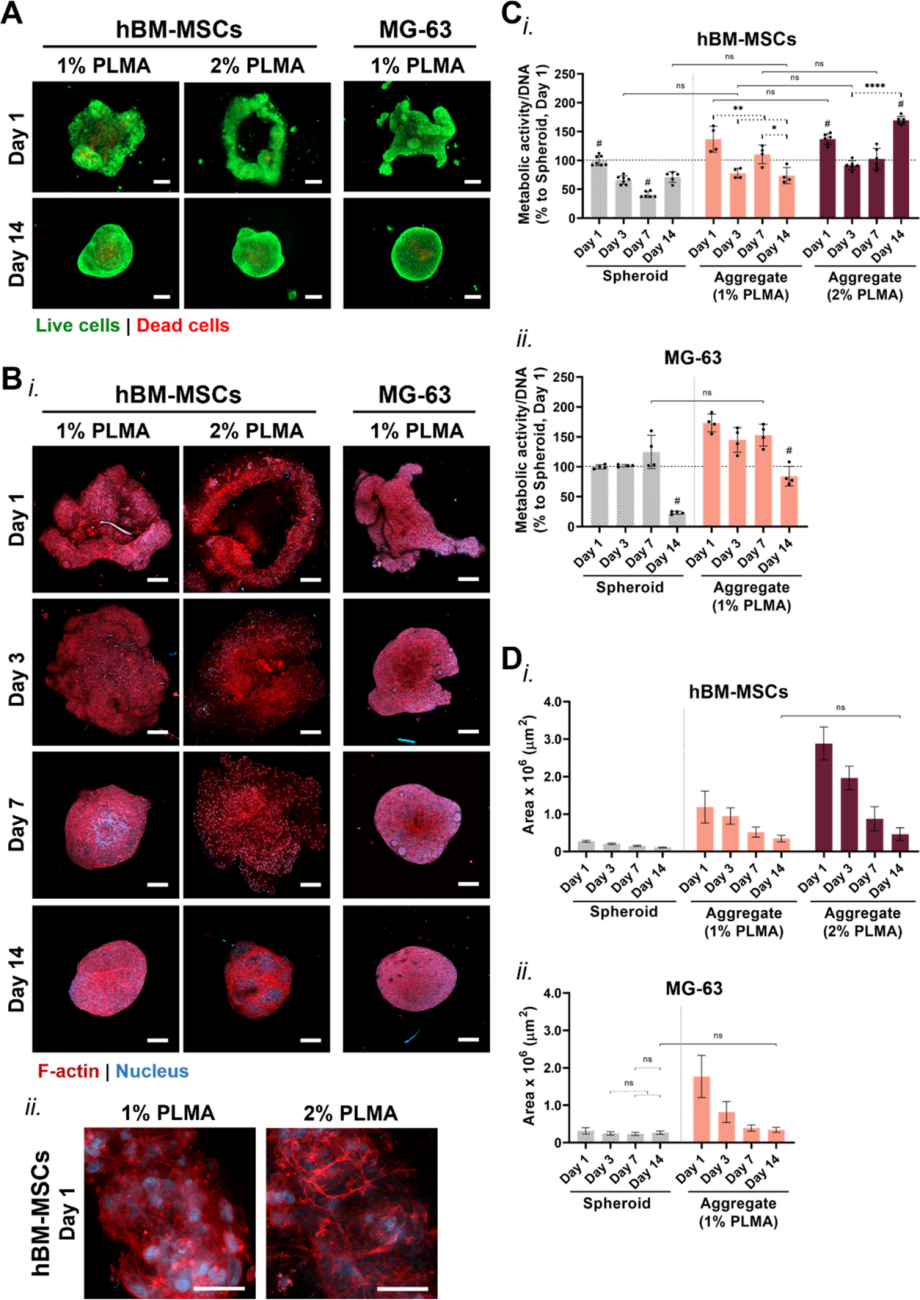
Viability and morphological characterization of hBM-MSCs and MG-63 aggregates integrating the PLMA fibril-derived membrane. (A) Live/dead images of hBM-MSCs and MG-63/membrane aggregates after 1 and 14 days in culture. Scale bar: 200 μm. (B) Confocal laser microscopy images of the (i) cell/membrane aggregates over 14 days in culture (scale bar: 200 μm) and (ii) hBM-MSCs stretched on top of the membrane at day 1 (scale bar: 50 μm). (C) Cell metabolic activity of (i) hBM-MSCs and (ii) MG-63 spheroids and aggregates formed with the PLMA fibril membrane, cultured for up to 14 days. Metabolic activity was normalized to DNA content and the data are represented as a percentage of the metabolic activity/DNA of the spheroid on day 1 of culture. Data are presented as mean ± SD (*n* ≥ 3). Statistical significance within the same model is represented with a dashed line. Comparison between conditions for the same time point is statistically significant, unless represented with ns (not significant). *p < 0.05; **p < 0.01; ****p < 0.0001. # represents significant difference to the remaining conditions at the same time point. (D) Area of the spheroids and cell aggregates measured over time. Data are presented as mean ± SD (*n* ≥ 3). Statistical significance within the same model is represented with a dashed line. Comparison between conditions for the same time point is statistically significant, unless represented with ns (not significant).

In order to investigate the potential influence of membrane fibril quantity on aggregate compaction, PLMA fibril membranes were fabricated using fibril suspensions containing three different volumes (5, 7.5, and 10 μL) derived from 1 and 2% PLMA solutions. Given the main purpose of this work to generate 3D cell-mediated aggregates incorporating the PLMA fibril membranes, these low amounts of fibrils (0.05–0.20 mg) were chosen to ensure low membrane thickness and high flexibility suitable for cell-guided folding. As expected, the amount of fibrils used to fabricate the membranes only affected aggregate compactness for the hBM-MSCs, with no impact on cell viability (Figure S6). While minor differences were observed for the different aggregates generated with membranes fabricated from the 1% PLMA solution, the self-aggregation of hBM-MSCs cells differed when employing membranes fabricated from the 2% PLMA solution. The membranes formed with 5 μL of fibril suspension gave rise to loosening and fragile aggregates constituted by some cell agglomerates, whereas membranes containing twice the fibril quantity resulted in uncompleted cell/membrane aggregation. To compare these cell aggregates with traditionally generated cell spheroids, membranes fabricated with a volume of 7.5 μL of a fibril suspension were selected for the following experiments. Moreover, since no relevant differences were observed for the MG-63-guided aggregation, further analysis with these cells was limited to the membranes produced from 1% PLMA fibril solution. Results revealed that both cell/membrane aggregates progressively compacted throughout the 14 days of culture, autonomously organizing in regions with higher and lower cell density (Figure S7A,B). Compared with the traditional cell spheroid, both cell/membrane aggregates showed superior cell viability with minimal or no necrotic core formation (Figures 5A and S8A). Interestingly, the amount of PLMA used to generate the fibril building blocks (1 vs 2% w/v PLMA) exhibited a pronounced effect on the viabilty of hBM-MSCs/membrane aggregates. Besides presenting fewer dead cells than their spheroid counterparts, the increase in membrane fibril content was reflected in the highest cell viability. Even though the formation of a necrotic core has been considered an important characteristic in studying advanced tumor stages, these regions of oxygen depletion can have a detrimental effect on stem cells.^74^ In this point of view, the strategy of cell aggregate formation herein proposed is promising to mitigate this phenomenon. Cell morphology analysis of the aggregates showed cell spreading on the fibril membrane and F-actin filament organization (Figure 5B), demonstrating the engagement of a mechano-sensitive cell integrin–ECM interaction that propels F-actin cytoskeleton organization.^75^ These images also revealed a densely interconnected network of elongated cells, progressing into more compact 3D structures with increased cell elongation over time, in contrast to cell spheroids (Figures 5B and S8B). In fact, cell/membrane aggregates resulted in increased cell metabolic activity at day 1 for both cell types under the studied conditions (Figure 5C). In accordance with live/dead staining results, both PLMA membrane-based hBM-MSC aggregates showed higher metabolic activity on day 7 compared to their spheroid counterpart. Moreover, the membrane fibril quantity seems to contribute to a significant increase in the metabolic activity of the hBM-MSCs/membrane aggregates over the 14 days in culture. Concerning the MG-63 tumor aggregates, the metabolic activity was higher in all time points when compared with their spheroid counterparts, with an accentuated difference at day 14 due to the lower necrotic core formation. Such an increase in cell metabolic activity is related to the lower compactness of the cell aggregates generated with the free-standing membranes, a phenomenon that is hypothesized to increase the nutrient availability in the core of the aggregates and consequently, the cell viability, as demonstrated by live/dead staining.

Due to the comparatively slower compaction of the membrane-supported cell aggregation as opposed to spheroid formation, the area of the aggregates was measured over time for different conditions (Figure 5D). Of note, the fibril-derived membrane without cells was also considered for area quantification, as represented in Figure S7C. Results showed a significantly higher area for the hBM-MSCs and MG-63 aggregates in comparison to the spheroid counterparts. Nevertheless, cell/membrane aggregate areas decreased gradually over time. It is worth noticing that, in the case of hBM-MSCs, the membrane fibril quantity also influenced the cell/membrane aggregate area, although resulting in aggregates with similar areas at 14 days of culture. The MG-63/membrane aggregates developed a 3D spherical structure with an area similar to a spheroid as a result of cells’ high compaction ability over time, even though with higher cell viability and metabolic activity. The higher area verified for the hBM-MSC aggregates formed with the 2% PLMA fibril membrane suggests that the cell-guided membrane folding is constrained, possibly due to an increase in membrane stiffness. As previously discussed, fibril membranes produced with increasing fibril content exhibit increased thickness (Figure 3D), which can be characterized by higher bending stiffness.^63,64^

**Figure 6:**
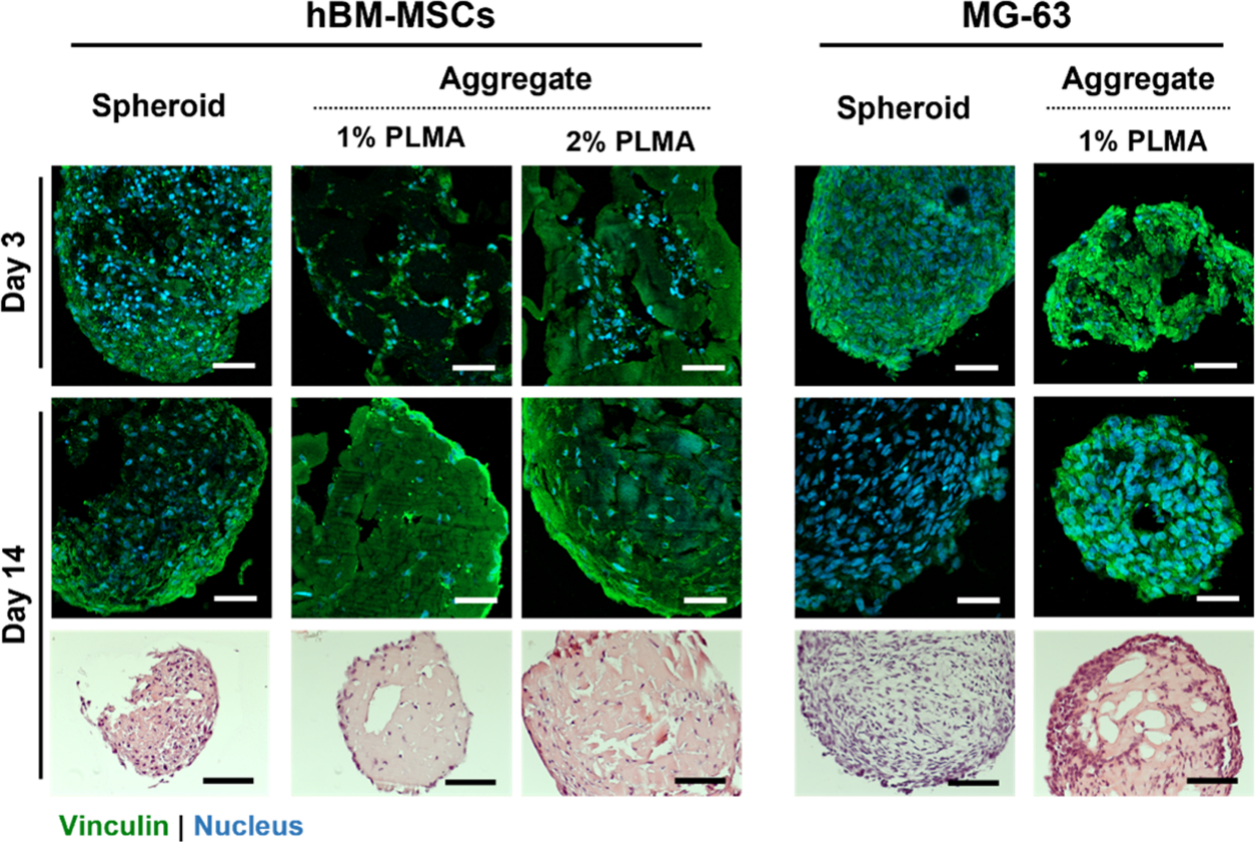
Vinculin expression and 3D microtissue organization. Immunohistochemistry of vinculin expression in the cell spheroids and aggregates formed with hBM-MSCs and MG-63, at 3 and 14 days of culture. Scale bar: 50 μm. Hematoxylin and eosin (H&E) staining of the studied spheroids and aggregates occurred at 14 days of culture. Scale bar: 100 μm.

Besides the organization of the F-actin filaments in the cell cytoskeleton, the expression of proteins such as vinculin that constitute the force transduction complex intermediating the integrin–ECM signaling and the cytoskeleton is essential to conclude about the maturity of cell–ECM adhesion and motility.^75^ As an important player in cell polarization and migration through cell–cell and cell–ECM interactions,^76^ vinculin staining in histological slices was found to be expressed by day 14 in both hBM-MSCs and MG-63/membrane aggregates (Figure 6). Notably, a decreased expression of vinculin in MG-63 spheroids was observed on day 14, in accordance with the low level of eosin staining. Vinculin expression has been associated with tumor progression and metastasis,^77^ highlighting the feasibility of the tumor aggregate to better reproduce the tumor microenvironment. Indeed, the live imaging experiment revealed the high motility ability of the tumor cells cultured on the membrane (Supporting Movie 5). Additionally, the histological examination of spheroids and aggregate microtissues revealed that aggregates facilitated the formation of more extensive matrix-rich regions surrounding individual cells or cell agglomerates (Figure 6). Interestingly, these regions constituted by the PLMA fibril membrane resemble the ECM regions surrounding cell agglomerates observed in native tissues.^32^ The present observation validates the cell accumulation on the outer limits of the aggregates observed in the live imaging experiment and is attributed to the cell-guided membrane folding phenomenon. Interestingly, the reduced cell aggregation observed in the initial days of culture using the 2% PLMA fibril-derived membrane reflected in a better distribution of the cells within the aggregate internal. Moreover, the presence of numerous void spaces can contribute to increased nutrient availability in the interior of the aggregates, positively influencing cell viability over time.^78^ In fact, cell spheroids demonstrate high compactness from day 1, which can limit nutrient diffusion, while cell/membrane aggregates present void spaces throughout the 14 days of culture (Figures S7 and S9). Altogether, the self-aggregation of cells on a thin PLMA fibril-derived membrane rendered 3D spheroid-like microtissues that not only enhanced cell viability but also facilitated cell–cell and cell–ECM interactions, exhibiting a tissue organization similar to native tissues.

## Conclusions

Amyloid-like fibrils have been considered promising bioactive supramolecular materials for tissue engineering purposes, owing to their role in several physiological processes. By exploiting a human source of proteins rich in growth factors, platelet lysate (PL), a simple and reproducible strategy of generating 3D self-assembled microtissues incorporating human-derived free-standing fibril membranes, is herein described. The PL- and PLMA-derived amyloid-like fibrils were efficiently and instantaneously produced, leveraging the capability of ionic liquid (IL) [Cho][TOS] to promote protein fibrillation. As building blocks for the generation of free-standing membranes, the protein fibrils rendered thin, flexible, and mechanically tunable membranes with nanotopographical cues that facilitated cell adhesion. Triggered by the free cell movement and high traction forces, cell-guided membrane folding induced the formation of 3D spheroid-like microtissues. Besides improving cell viability and metabolic activity, this strategy of forming 3D cell aggregates enhanced the cell–matrix interactions, resulting in a native-like microtissue organization constituted by ECM-rich areas surrounding cell regions. From a biomedical point-of-view, a more realistic recreation of the tissues’ organization with the cell self-aggregated 3D microtissues can contribute to a better recapitulation of the in vivo cell behavior. Owing to the ability to recreate the tumor architecture, this biomimetic platform can contribute with significant insights into comprehending tumor progression. Moreover, the stem cell spreading and viability in these self-assembled aggregates are anticipated to provide cutting-edge cell therapeutic agents for clinical tissue injuries. Overall, the development of 3D microtissues by cell self-aggregation incorporating a free-standing PLMA fibril membrane was revealed to be a promising strategy to advance disease modeling and foster the establishment of cell carriers for a wide range of biomedical applications and following the animal-free tendency.

## Methods

### Preparation of Human Platelet Lysate-Derived Proteins

Human platelet lysate (PL, STEMCELL Technologies, Canada) was isolated from the peripheral blood of healthy donors, under licensing by the US Food and Drug Administration (FDA) and Health Canada. Samples from multiple donors (>100 donors) were pooled to minimize lot-to-lot variability. It is worth noticing that no additional ethical approval was required as the platelet lysate was acquired from a third-party supplier. Prior to use, frozen platelet lysate supplied with a protein content of 55–65 mg·mL^–1^ was thawed in a 37 °C water bath and aliquoted. Liquid platelet lysate was freeze-dried (LyoAlfa 15, Telstar) and stored at 4 °C. Methacryloyl platelet lysate (PLMA, Metatissue, Portugal) of human origin was acquired and stored at 4 °C until further use, following manufacturer’s instructions. It is worth noting that PLMA was synthesized from human PL from the same manufacturer as the PL used in this work (STEMCELL Technologies).

### Quantification of Chemically Modified Amines

The degree of methacrylation of PLMA amines was estimated through the 2,4,6-trinitrobenzenesulfonic acid (TNBSA) assay. A reaction buffer constituted of 0.1 M sodium bicarbonate (Sigma-Aldrich) at pH 8.5 was used to dissolve the lyophilized PL and PLMA samples. The TNBSA reagent (Fisher Scientific) at a concentration of 0.01% (w/v) was added to the samples, and the samples were incubated at 37 °C for 2 h. To stop the reaction, a solution of 10% (w/v) SDS (Sigma-Aldrich) and 1 M hydrochloric acid (HCl, Sigma-Aldrich) was added. Afterward, the absorbance was read at 650 nm in a Synergy HTX microplate reader (BioTek Instruments, Winooski). The degree of modification of the amines was quantified according to the following eq 1:
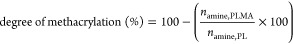
1

### Ionic Liquid Synthesis

The ionic liquid (IL) cholinium tosylate ([Cho][TOS]) was synthesized through an acid–base reaction based on a protocol reported elsewhere.^79^ Cholinium bicarbonate (80% in water with purity >98%, Proionic, Austria) and *p*-toluenesulfonic acid monohydrate (>98.5%, Alfa Aesar) were mixed in a 1:1 ratio and kept under constant stirring for 24 h at room temperature (RT). The reaction product was washed with ethyl acetate (99%, Carlo Erba, Germany), and volatile solvents were removed in a rotary evaporator for 1 h at 60 °C. Then, the [Cho][TOS] was dried under a high vacuum at 70 °C for 48 h, and the obtained white solid was stored in a vacuum desiccator until further use.

### Preparation of PL and PLMA Protein Fibrils

PL and PLMA solutions at 1 and 2% (w/v) were prepared in sterile ultrapure water from the freeze-dried materials and acidified to pH 2 with 1 M HCl. Protein fibrils were instantaneously formed by adding 15 μL of a 1 M [Cho][TOS] sterile solution in ultrapure water to 100 μL of PL/PLMA protein solution, followed by vigorous agitation in a vortex and incubation for 15 min at RT.

### Fibril Morphology Characterization

Freshly prepared PL and PLMA fibril morphology was characterized by imageology techniques by resorting to optical microscopy, confocal fluorescence microscopy, and transmission electron microscopy (TEM).

#### Confocal Microscopy

Protein fibrils were stained in a solution of 1:100 of fluorescein-5(6)-isothiocyanate (FITC, 1 mg·mL^–1^ in distilled water, Sigma-Aldrich) for 1 h. Samples were centrifuged and washed three times with ultrapure water, mounted in an 8-well plate (ibidi, Germany), and observed under a confocal microscope (LSM 900, Carl Zeiss, Germany) using an oil-immersion 60× lens.

#### TEM

Freshly prepared protein fibrils were fixed with a solution of formaldehyde (4% (v/v), Sigma-Aldrich), washed with distilled water (phosphate-buffered saline (PBS), Sigma-Aldrich), and stained with uranyl acetate for 1 h. The samples were transferred to a carbon-film-supported copper grid, dried overnight at RT, and analyzed using TEM. TEM analyses were performed using a JEOL 2200FS (JEOL, Japan) transmission electron microscope operating at an acceleration voltage of 200 kV.

### Circular Dichroism (CD) Spectroscopy Analysis

Protein fibrils from PL and PLMA solution (1% (w/v)) were prepared as described above and washed three times with ultrapure water through centrifugation. Fibrils were resuspended in ultrapure water and dispersed via sonication for 5 min. In order to analyze the protein conformational transition, native PL/PLMA protein solutions were also prepared for CD analysis. Protein and fibril solutions were properly diluted to ensure a spectral HT value below 600 V, and the CD spectra were recorded at RT in the UV region (190–260 nm), using a J-815 CD spectrometer (Jasco, Japan). Spectra were acquired in a 1.0 cm path length quartz cuvette at a scan rate of 100 nm·min^–1^ and a data pitch of 0.5 nm.

### Fourier Transform Infrared (FTIR) Spectroscopy Analysis

PL and PLMA fibrils were freshly prepared as described previously, centrifuged, washed with ultrapure water, and air-dried overnight. The resulting material was used to evaluate the secondary structure along with lyophilized PL and PLMA proteins. FTIR spectra were acquired in a wavelength range from 1700 to 1200 cm^–1^ in a GALAXY SERIES FTIR 7000 (Mattson Instruments). Under controlled humidity, 256 scans were carried out for each sample with a resolution of 2 cm^–1^.

### Thioflavin T (ThT) Fluorescence Assay

The dependency of protein β-sheet structure formation on [Cho][TOS]–protein interaction was evaluated using thioflavin T (ThT, Sigma-Aldrich), a molecule that emits a fluorescence signal when intercalating with protein β-sheet structures. The acidified protein solutions (PL and PLMA at 1 and 2% (w/v)) were diluted in a 1:8 ratio in distilled water, and 10.4 nmol of ThT solution (10 mM) was added. Different aliquots of [Cho][TOS] were further added to each sample, and the resulting interactions were analyzed by measuring the ThT fluorescence in a quartz cuvette. The protein solution with ThT was used as a baseline, and the intrinsic fluorescence of the protein solutions (without ThT reagent) was measured for comparison purposes. The spectra were recorded in an FP-8300 fluorometer (JASCO, Japan) between 420 and 600 nm under an excitation wavelength of 412 nm. Excitation and emission spectra bandwidths were set to 5 nm, and the data pitch was set to 0.5 nm. The emission spectrum of the ThT solution in water was used as the baseline, and the fluorescence emission spectrum of the diluted protein with the highest concentration of [Cho][TOS] was acquired for comparison.

### Fibril Formation Efficiency

#### Protein Quantification (Indirect)

The efficiency of protein fibrillation was indirectly evaluated by quantifying the amount of protein that contributed to fibril formation using the Bradford assay kit (Thermo Fisher Scientific), according to the manufacturer’s recommendations. Different molecular concentrations of [Cho][TOS] (ranging from 0.5 to 30 μmol) were added to acidified PL/PLMA solutions at 1 and 2% (w/v), vigorously vortexed, and centrifuged at 15,000 rpm for 15 min. The supernatants were collected and used to quantify the proteins that did not contribute to fibril formation. Low-binding materials have been used to reduce the protein adhesion. It is worth noting that this quantification may be overestimated because of indirect quantification. The percentage of fibrillated protein was calculated based on eq 2:
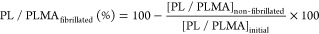
2where PL/PLMA_fibrillated_ is the percentage of protein that contributed to fibril formation, and [PL/PLMA]_nonfibrillated_ and [PL/PLMA]_initial_ are the protein concentration quantified in the supernatants and in the initial protein solutions, respectively.

#### Sodium Dodecyl Sulfate Polyacrylamide Gel Electrophoresis (SDS-PAGE)

The protein profiles of each fibril sample (PL and PLMA) were evaluated by sodium dodecyl sulfate polyacrylamide gel (SDS-PAGE). The fibrils were destroyed by adding 2-mercaptoethanol (ME, 66 μL, Alfa Aesar) to the suspension. For the controls (PL and PLMA), ME was also added to the protein solution. The amount of protein in each sample was assessed using the Bradford assay (Thermo Fisher Scientific), following the manufacturer’s instructions. The samples were then subjected to sodium dodecyl sulfate polyacrylamide gel electrophoresis. Previously, destroyed fibrils were diluted in sample buffer (100 mM Tris–HCl (Sigma-Aldrich) at pH 6.8, 4% sodium dodecyl sulfate (SDS, Sigma-Aldrich), 20% glycerol (99%, Sigma-Aldrich), and 200 mM ME) and heated at 85 °C for 5 min. Denatured samples (10 μg per well) were then loaded in 4–12% Tris–glycine (Novex WedgeWell, Invitrogen), and the protein profile was evaluated against the controls.

### Formation of Protein Fibril-based Free-Standing Membranes

Considering the results obtained from the previous structure and conformational characterization, as well as the fibrillation efficiency, only PLMA fibrils were used from now on. The suspension of PLMA fibrils prepared as described above was used to fabricate thin protein fibril-based membranes by solvent casting. Briefly, a defined volume (5, 7.5, or 10 μL) of PLMA fibril suspension was pipetted into a μ-Slide Angiogenesis plate (ibidi, Germany) and dried on a 37 °C hot plate for 1 h. The casted membrane was rehydrated with distilled water, and membrane detachment from the surface was observed by simply flipping the plate and imaged by optical contrast microscopy. The solvent-casting process of membrane formation was observed by producing PLMA fibril membranes on a superhydrophobic–superhydrophilic microarray fabricated as reported elsewhere.^80^

To analyze the membrane structure, freshly prepared PLMA membranes were fixed with carbon tape on a stub and spin-coated with a thin layer of palladium gold. Images were captured using a Hitachi SU-70 scanning electron microscope (SEM, Hitachi, Japan) with an acceleration voltage of 4 kV. To measure membrane thickness, PLMA fibril membranes prepared with different fibril contents were placed on carbon tape and imaged using a Hitachi SU-3800 SEM (Hitachi, Japan) with an acceleration voltage of 5 kV.

### Membrane Stability

To assess the membrane stability for cell culture purposes, membranes formed with 7.5 μL of PLMA fibril suspension were rehydrated in Minimum Essential Medium α (α-MEM, Thermo Fisher Scientific) supplemented with sodium bicarbonate (2.2 g·mL^–1^, Sigma-Aldrich), 10% (v/v) heat-inactivated fetal bovine serum (FBS, Thermo Fisher Scientific), and 1% (v/v) antibiotic/antimycotic (10,000 units·mL^–1^ penicillin, 10,000 μg·mL^–1^ streptomycin, and 25 μg·mL^–1^ amphotericin B, Thermo Fisher Scientific). Membranes were incubated in a humidified incubator under cell culture conditions (5% CO_2_, 37 °C); the medium was changed every 2 to 3 days; and they were observed by optical contrast microscopy.

### Protein Release from the Membranes

The release of proteins from the PLMA membrane (1 and 2% (w/v)) was evaluated by incubating the membranes in α-MEM medium without FBS, in tubes (500 μL, 3 membranes per tube, *n* = 6 per condition), under cell culture conditions. Throughout the 14 days of the experiment, 250 μL of the supernatant was collected at each time point, and fresh cell culture medium (250 μL) was added. Aliquots were stored at −80 °C until further use. For released protein quantification, the Bradford assay kit was used following the manufacturer’s instructions.

### Cell Culture

MG-63 cell line (European Collection of Authenticated Cell Cultures, ECACC, U.K.) and human bone marrow mesenchymal stem cells (hBM-MSCs, American Type Culture Collection, ATCC) were cultured in α-MEM cell culture medium. The cells were maintained under standard cell culture conditions (humidified atmosphere with 5% CO_2_ at 37 °C). hBM-MSCs in cell passages 4–7 were used in the experiments.

The tumor cell line MG-63 was transfected with lentivirus expressing a red fluorescent protein (CMV-RFP, Cellomics Technology). Cells were cultured overnight in a 24-well plate and then transfected at a multiplicity of infection (MOI) of 10 for 12 h in α-MEM cell culture medium containing polybrene (6 μg·mL^–1^, Sigma-Aldrich). Protein-expressing cell selection was performed 3 days after transfection with α-MEM supplemented with puromycin (2 μg·mL^–1^, STEMCELL Technologies).

### Cytotoxicity of PLMA Fibrils

MG-63 and hBM-MSCs were seeded overnight on 96-well plates (10,000 cells per well) and then incubated for 3 days in cell culture medium containing different amounts of suspended PLMA fibrils (1 and 2% (w/v)) in a range of 0–4.34 μg. To ensure that the ionic liquid [Cho][TOS] does not affect cell viability by itself, a similar experiment was performed by culturing the cells in a cell culture medium with [Cho][TOS] in the range of 0–5.0 μmol. The different amounts of [Cho][TOS] correspond to the quantity of IL in the PLMA fibril solution added to study the cell viability in the presence of the protein fibrils. After 3 days of culture, cell viability was assessed by incubating the cells for 4 h with the AlamarBlue reagent (Invitrogen) at a ratio of 1:10 in α-MEM. Resazurin fluorescence was measured in a 96-well flat-bottom opaque black plate by using a microplate reader.

The maintenance of cell morphology in culture with PLMA fibrils was addressed by seeding the cells overnight in 8-well plates (20,000 cells per well, ibidi) and then culturing them with 5 μL of protein fibril suspension. On day 3, cells were washed with Dulbecco’s phosphate-buffered saline (dPBS) and fixed in a 4% formaldehyde solution for 30 min. Afterward, the samples were permeabilized with 0.5% (v/v) Triton X-100 (Sigma-Aldrich) for 5 min and stained for the actin filaments with 1:8 Phalloidin-iFluor 594 reagent (ab176757, Abcam, U.K.) solution in PBS for 1 h at RT, followed by nucleus staining with 1:200 4′,6-diamidino-2-phenylindole (DAPI, Thermo Fisher Scientific) solution in PBS for 10 min at RT. After being washed with PBS, cells were observed under a confocal microscope.

### Generation of PLMA Membrane-Integrating Cell Aggregates

Different PLMA fibril suspension volumes (5, 7.5, and 10 μL) obtained from PLMA solutions at 1 and 2% (w/v) were used to produce PLMA fibril-based membranes by solvent casting on a μ-Slide Angiogenesis plate (ibidi), as above-described. The cells were detached with TrypLE Express (Gibco, Thermo Fisher Scientific), resuspended in the appropriate cell culture medium at a cell density of 0.6 million cells·mL^–1^ and then carefully pipetted onto the dried PLMA fibril membrane (50 μL per well, correspondent to 30,000 cells per membrane). To serve as a control for comparison analysis purposes, MG-63 and hBM-MSCs spheroids at a density of 30,000 cells were generated in 96-well round-bottom ultralow attachment plates (Corning, Thermo Fisher Scientific) without a centrifugation step. Both membrane-based cell aggregates and spheroids were maintained in culture for 14 days with medium change every 2 to 3 days and imaged by optical contrast microscopy in an inverted light microscope. Sample images were processed in ZEN Image software using the Image Processing tool.

### Real-Time Live Cell Tracking

MG-63 tumor cells transfected with RFP were seeded on a PLMA fibril-based membrane formed with 7.5 μL of fibril suspension from a 1% (w/v) PLMA solution. Before membrane formation, PLMA fibrils were stained with FITC as described earlier. The dynamics of cell aggregation with the support of the PLMA fibril membrane was followed by real-time imaging in a confocal microscope coupled with an incubation stage and a chamber. Time-lapse images were acquired every hour for 72 h and at 5, 7, and 14 days of culture.

### Cell Viability and Proliferation Analysis

The viability of spheroids and PLMA membrane-integrating cell aggregates was assessed at different time points by live and dead staining and metabolic activity quantification. At the specified time points, the samples were stained in a solution of 1:100 of Calcein AM solution (1 mg·mL^–1^ in dimethyl sulfoxide, Thermo Fisher Scientific) and 1:200 of propidium iodide (1 mg·mL^–1^ in distilled water, Thermo Fisher Scientific) in cell culture medium at standard cell culture conditions. The samples were observed under a wide-field microscope (Axio Imager M2, Carl Zeiss, Germany). To evaluate cell metabolic activity under different conditions, the CellTiter-Glo 3D Cell Viability Assay (Promega, Madison) was used as recommended by the manufacturer. Briefly, the samples were washed with dPBS and incubated in 0.25% trypsin/ethylenediaminetetraacetic acid (EDTA) (Gibco, Thermo Fisher Scientific) for 1 h to promote spheroid/aggregate disassembly. Afterward, cell culture medium and CellTiter-Glo 3D reagent were added to the trypsin cell suspension at a 1:1:2 ratio (trypsin/medium/reagent), and the samples were vigorously mixed for 5 min, followed by a 25 min incubation at RT. Lastly, a 96-well flat-bottom opaque white plate was used to measure the luminescence in a microplate reader. The samples were stored at −80 °C for DNA quantification.

Spheroid and aggregate proliferation was analyzed by DNA quantification using the Quant-iT PicoGreen dsDNA Assay Kit (Thermo Fisher Scientific), according to the manufacturer’s recommendations. After the samples were thawed at 37 °C, the DNA was quantified according to the manufacturer’s recommendations, using a 96-well flat-bottom opaque black plate. The fluorescence was measured after 10 min of incubation in a microplate reader at an excitation/emission wavelength of 480/528 nm.

### Morphological Characterization of Cell Spheroids and Aggregates

At different time points, spheroids and aggregates were washed with dPBS, fixed in a 4% formaldehyde solution for 2 h, and permeabilized with 0.5% (v/v) Triton X-100 for 30 min. The F-actin filaments and nucleus were then stained with 1:8 Phalloidin-iFluor 594 reagent (ab176757, Abcam) solution in PBS for 2 h at RT, followed by 1:200 DAPI (4′,6-diamidino-2-phenylindole, Thermo Fisher Scientific) solution in PBS for 30 min at RT, respectively.

The images acquired over time by optical contrast microscopy were used to measure the area and circularity of the spheroids and aggregates. Matlab (version R2022b, MathWorks Inc.) was used to run the high-throughput image analysis software SpheroidSizer, which applies an algorithm to measure spheroid size.^81^ The automatic and manual contour selections allow accurate delimitation of the spheroid/aggregate area. It is important to note that membrane regions not covered by cells in the first time points were also considered as part of the aggregate.

### Immunohistological Staining

Spheroids and aggregates collected after 3 and 14 days of culture were fixed as above-mentioned and embedded in HistoGel (Thermo Fisher Scientific). The samples were dehydrated in increasing grades of ethanol (Carlo Erba), cleared with xylene (Carlo Erba), and embedded in paraffin (Thermo Fisher Scientific). A microtome (HM 340E Electronic Rotary Microtome, Thermo Fisher Scientific) was used to cross-section the paraffin-embedded samples and the 5 μm sections were then deparaffinized in xylene and rehydrated. Hematoxylin and eosin (H&E) staining was performed by immersing the samples in Mayer’s hematoxylin (Sigma-Aldrich) solution for 8 min, followed by washing in distilled water and immersion in eosin Y (Sigma-Aldrich) for 1 min. Lastly, the samples were dehydrated, cleared, and mounted (DPX Mountant medium, Sigma-Aldrich) by posterior visualization in a wide-field microscope. To assess vinculin expression by immunohistochemistry, samples’ sections were submitted to antigen retrieval in 10 mM sodium citrate buffer (Sigma-Aldrich) at 95–100 °C for 20 min. After cooling down, the sections were permeabilized with 0.5% Triton X-100 for 5 min and blocked with 5% FBS (v/v) in PBS for 30 min. Afterward, the samples were incubated overnight in a humidified chamber at 4 °C with the monoclonal primary antibody rabbit antihuman vinculin (1:1000 in 5% FBS (v/v) in PBS, Thermo Fisher Scientific). The secondary antibody antirabbit AlexaFluor 488 (1:200 in 5% FBS (v/v) in PBS, Abcam) was then incubated for 1 h at RT, and the samples were counterstained with DAPI (1:1000) for 5 min. The samples were observed in a confocal microscope.

### Statistical Analysis

All data were statistically analyzed using GraphPad Prism 8 software (Dotmatics), using one-way analysis of variance (ANOVA) with Tukey’s multiple comparison tests. Data are expressed as mean ± standard deviation (SD). For *p*-values <0.05, data were considered statistically different and were represented by *p < 0.05, **p < 0.01, ***p < 0.001, and ****p < 0.0001.

## Supporting Information Available

Fibrillation process representation; FTIR spectra and amide I region deconvolution; PLMA amine content; IL-dependent fibril formation; SDS-PAGE of original protein solution and respective fibrils; contrast images of PLMA membranes; toxicity data for cell culture in the presence of PLMA fibrils and respective F-actin filaments and nuclei staining; microscopy images of cell-induced fibril membrane assembly; optical contrast, confocal live/dead and F-actin/nucleus, and histological H&E images of cell aggregates; and table of PL proteins secondary conformation and amino acid content (PDF)
Instantaneous PL protein fibrillation upon IL addition (Movie 1) (MP4)
Instantaneous PLMA protein fibrillation upon IL addition (Movie 2) (MP4)
Solvent-casting drying process of the PLMA fibril membrane (Movie 3) (MP4)
Hydrated PLMA fibril membrane (Movie 4) (MP4)
Cell stretching on top of the fibril membrane and consequent cell-mediated membrane folding (Movie 5) (MP4)
