# Supplementary material for: Human Platelet Lysate-Derived Nanofibrils as Building Blocks to Produce Free-Standing Membranes for Cell Self-Aggregation
Source: ACS Nano. 2024 Jun 4;18(24):15815–30. doi: 10.1021/acsnano.4c02790 (PMC11191744; doi:10.1021/acsnano.4c02790)
Supplement: Supplementary file 1 — nn4c02790_si_001.pdf [file nn4c02790_si_001.pdf]

Human Platelet Lysate-Derived Nanofibrils as Building Blocks to Produce Free-Standing Membranes for Cell Self-Aggregation

Cátia F. Monteiro[1], Maria C. Gomes[1], Pankaj Bharmoria[1†], Mara G. Freire[1], João A. P. Coutinho[1], Catarina A. Custódio[1], João F. Mano[1]**

[1] CICECO – Aveiro Institute of Materials, Department of Chemistry, University of Aveiro, Campus Universitário de Santiago, Aveiro 3810-193, Portugal

Supporting Information

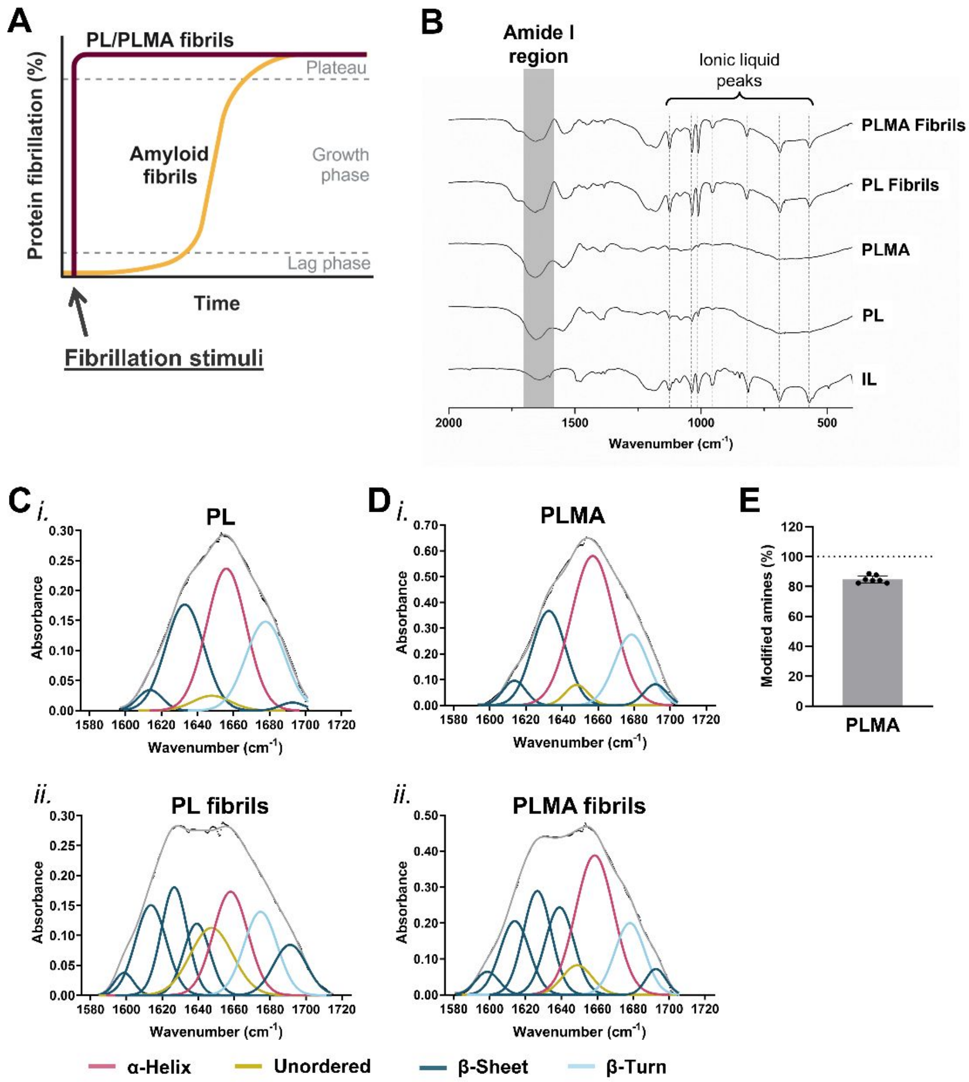

Figure S1. Characterization of platelet lysate-derived fibrils. (A) Representation of the fibrillation process for the native amyloid fibrils and the PL/PLMA fibrils instantaneously formed by the addition of IL. (B) Fourier Transform Infrared (FT-IR) spectra of IL, original protein sources (PL and PLMA), and protein fibrils. The amide I region used to characterize the protein secondary structure by deconvolution is highlighted in grey. Peaks identified in the protein fibrils and present in the IL spectra are identified with vertical dashed lines. (C,D) Gaussian deconvolution of the FT-IR spectra in the amide I region for the (i) original lyophilized PL and PLMA proteins and for the (ii) PL/PLMA fibrils. The experimental data represented as a dotted black line is overlapped by a solid grey line corresponding to the deconvolution fitting curve. The contribution of the secondary structures is shown as solid color lines. (E) Quantification of the chemically modified amines in the PLMA, compared to the total amine content in the PL (100 %). Data is presented as mean ± SD (n ≥ 3).

Table S1. Main PL proteins secondary structure and amino acid content. Representation of the conformational features and amino acid content in the most abundant PL proteins[1], indicating the number of the secondary structures and the percentage (%) of amino acids involved in those structures. Amino acids not contributing for the secondary structures presented in the table, are attributed to unordered structures. The most abundant secondary structures in terms of number and amino acids involved are highlighted in blue. The UniProt database was used to collect the data presented.

Protein	Nº of amino acids	% Hydrophobic amino acids	Nº of Secondary structures			% of Amino acids in secondary structures		
			α-Helix	β-strand	Turn	α-Helix	β-strand	Turn
**Serum albumin**	609	53.53	34	14	8	68.31	7.22	4.43
**Serotransferrin**	698	50.29	37	42	10	33.24	27.36	5.16
**Immunoglobulin gamma-1 heavy chain**	449	55.01	13	40	5	9.35	51.45	3.34
**Immunoglobulin heavy constant gamma 2**	395	56.96	5	18	0	5.82	36.71	0.00
**Complement C3**	1663	53.04	45	110	21	21.41	43.48	4.21
**Complement C4-B**	1744	54.99	13	68	2	4.42	26.55	0.52
**Actin, cytoplasmic 1**	375	58.40	21	16	5	39.20	21.60	4.00
**Immunoglobulin heavy constant alpha 1**	398	64.07	4	20	4	5.03	23.62	3.27
**Complement factor H**	1231	45.41	6	105	3	1.87	38.02	0.97
**Immunoglobulin kappa constant OS**	107	47.66	2	9	3	10.28	57.01	8.41
**Keratin, type II cytoskeletal 1**	644	51.55	2	0	0	32.30	0.00	0.00
**Alpha-2-macroglobulin**	1474	54.48	3	18	1	1.09	8.07	0.20
**Haptoglobin**	406	53.45	7	23	6	8.87	30.30	5.17
**Prothrombin**	662	50.76	18	40	14	16.62	26.44	7.25
**Immunoglobulin heavy constant gamma 3**	446	58.97	5	19	0	5.16	24.89	0.00

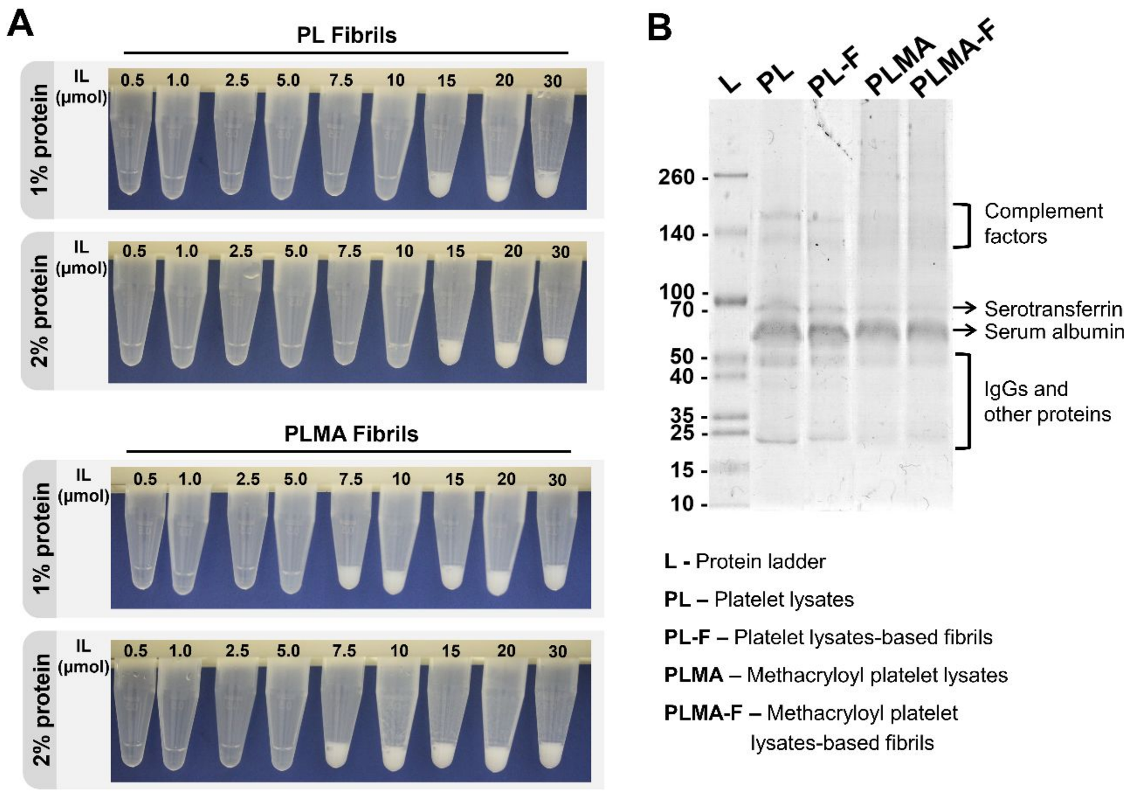

Figure S2. Efficiency of protein fibrillation. (A) Digital photographs of protein fibrillation in the presence of different amounts of the IL [Cho][TOS]. The whitish opaque solution results from the complete protein fibrillation. (B) Sodium dodecyl sulfate-polyacrylamide gel electrophoresis (SDS-PAGE) of the original protein solutions, PL and PLMA, and respective amyloid-like fibrils.

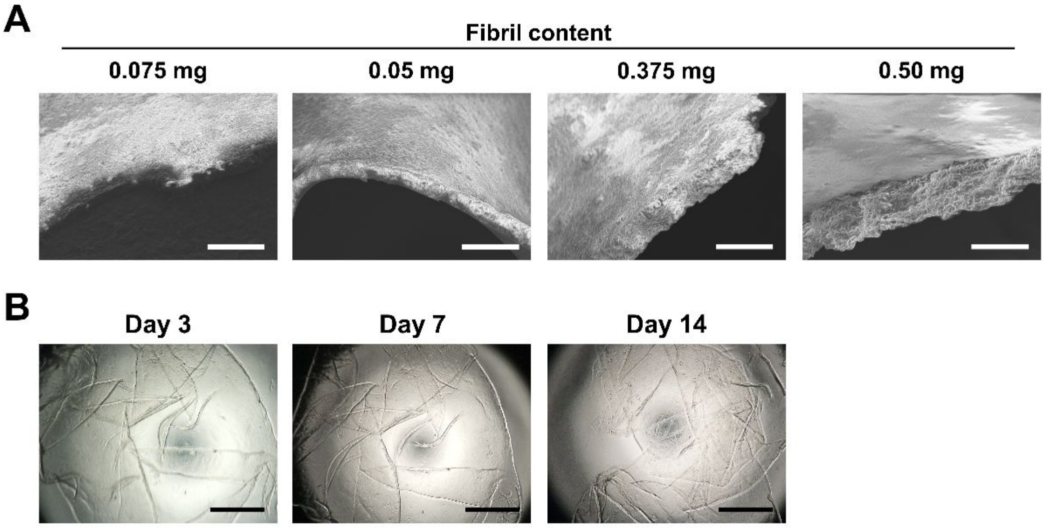

Figure S3. Fibril-based membrane thickness and stability. (A) Scanning electron microscopy images revealing the thickness of the PLMA fibril membranes produced with different fibril content. Scale bar: 40 µm. (B) Differential interference contrast images of the PLMA fibril membranes after 3, 7, and 14 days incubated under cell culture conditions. Membrane folds observed in all images result from the unintentional membrane movement during culture medium changes, due to the thickness and flexibility of the membrane. Scale bar: 500 µm.

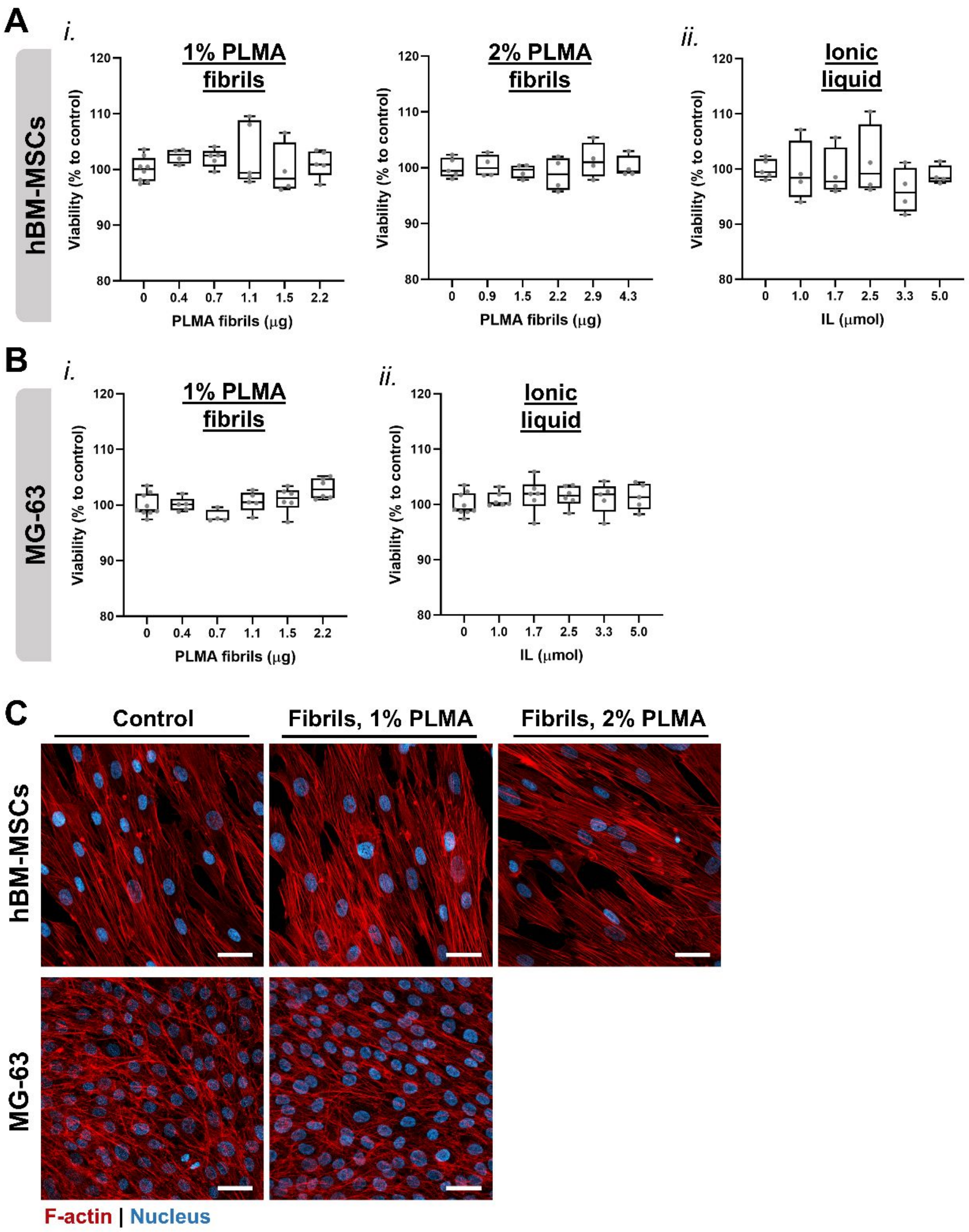

Figure S4. PLMA fibril biocompatibility. Viability quantification of (A) hBM-MSCs and (B) MG-63 cells cultured in 2D, exposed to different amounts of (i) PLMA fibrils produced from 1 and 2% (w/v) of protein solution. (A,B) (ii) Cell viability exposed to different quantities of IL, encompassing the IL content present in protein fibril suspension used to produce the fibrils. Data is presented as mean ± SD ($n \geq 3$) relative to control. (C) Confocal laser microscopy images of the 2D cell monolayers incubated for 3 days with the amount of protein fibrils corresponding to a membrane formed with 7.5 µL of fibril suspension. F-actin filaments and nuclei are stained in red and blue, respectively. Scale bar: 200 µm.

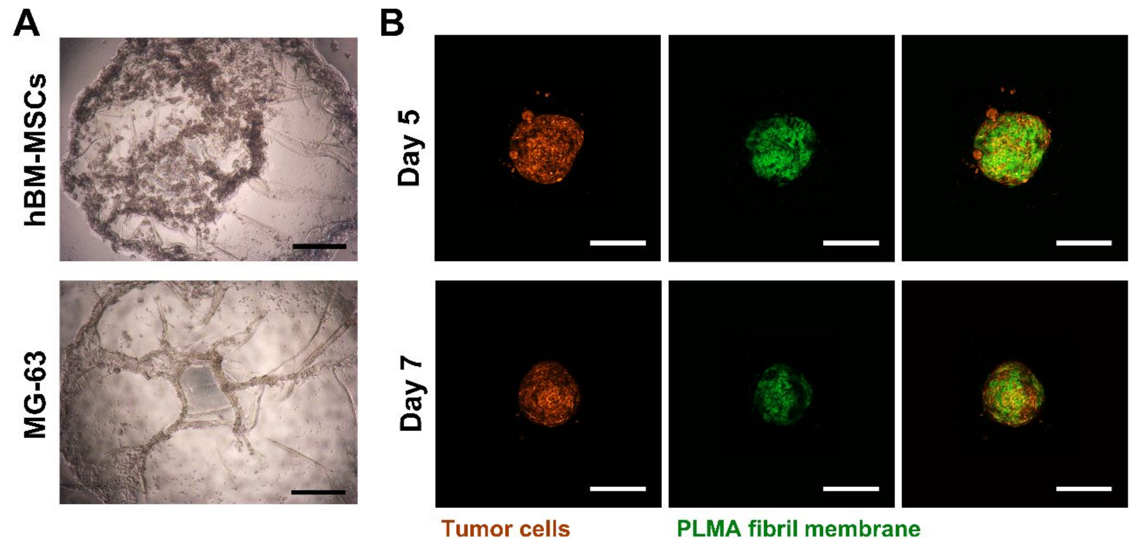

Figure S5. Cell-guided folding of the PLMA fibril membrane to generate 3D self-assembled aggregates. (A) Differential interference contrast microscopy images of hBM-MSCs and MG-63 cultured for 4 h on the top of a 1% PLMA fibril-derived membrane, forcing the formation of membrane folds. (B) Confocal laser microscopy images of the overall cell aggregate formed at 5 and 7 days in culture. Scale bar: 500 µm.

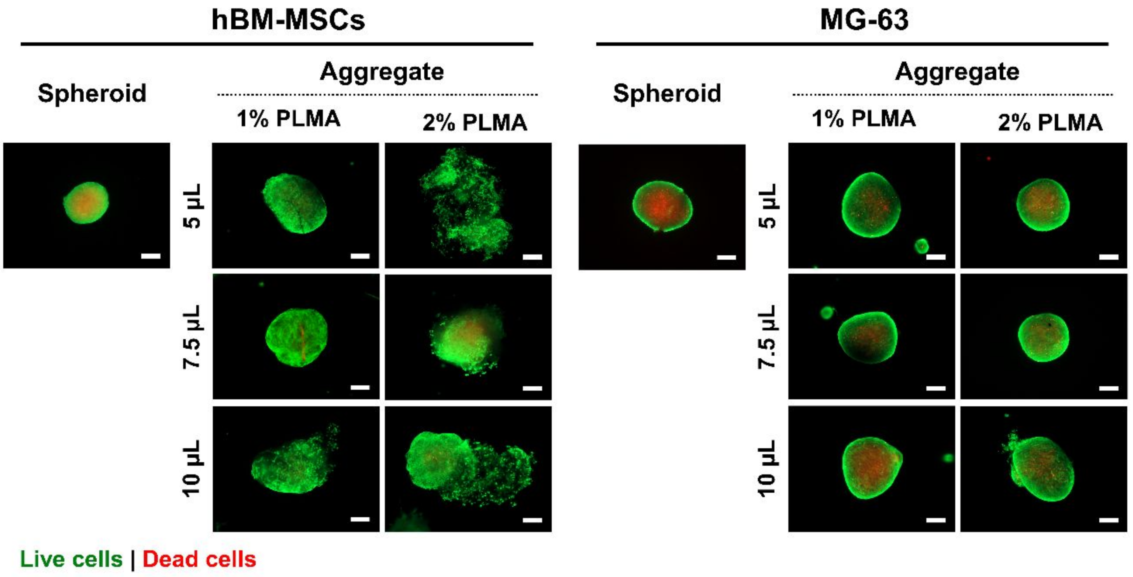

Figure S6. Cell self-aggregation dependence of membrane fibril quantity. Live/dead images of hBM-MSCs and MG-63 aggregates formed with fibril-based membranes with different fibril suspension volume (5, 7.5, and 10 µL) produced from 1 and 2% (w/v) of PLMA solution, after 14 days of culture. Spheroids generated in ultra-low adhesion U-shape plates were used as controls for comparison purposes. Scale bar: 200 µm.

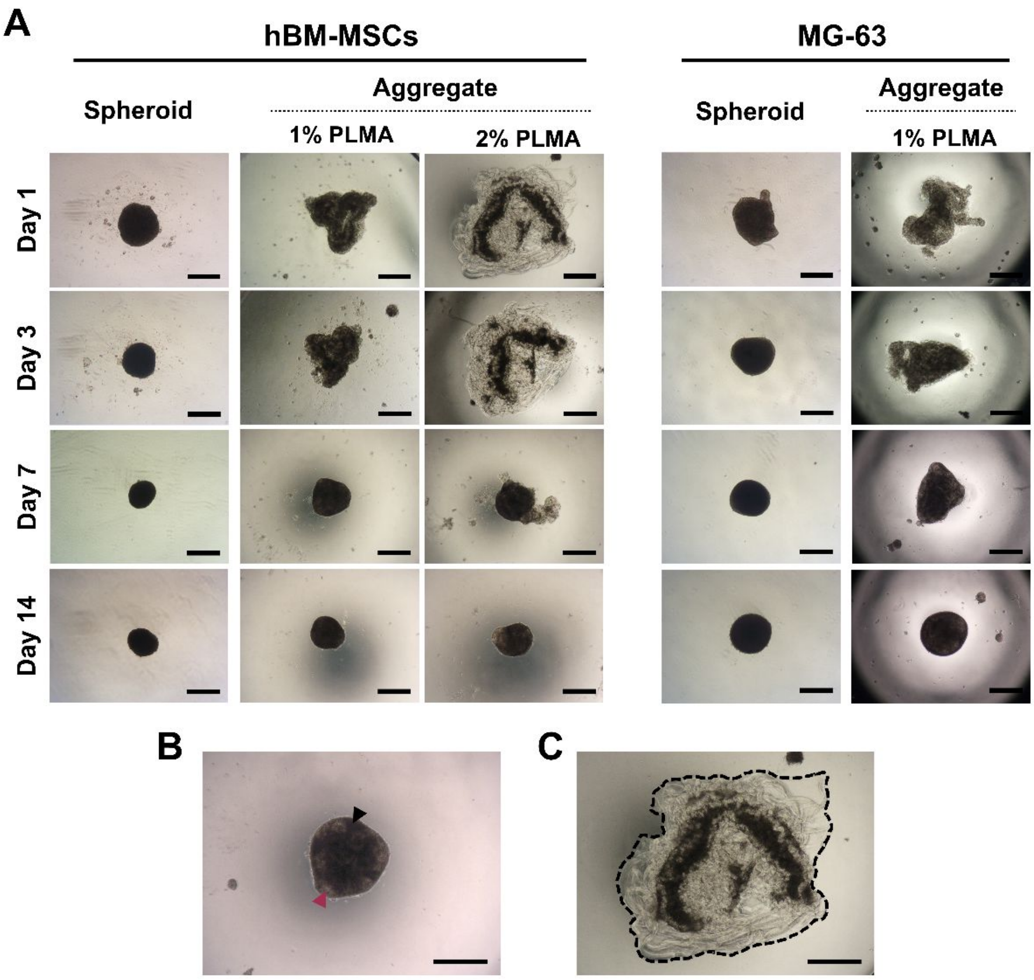

Figure S7. Comparison of spheroid and fibril-derived membrane cell aggregation. (A) Differential interference contrast microscopy images of hBM-MSCs and MG-63 spheroids and fibril-based membrane cell aggregates over 14 days in culture. (B) Representative image of a cell aggregate, indicating the presence of well-distributed high and low cell density in the aggregate (black and pink arrowhead, respectively). (C) Representative image of a cell aggregate cultured for 1 day demonstrating the strategy used to measure the area of the cell aggregates, considering the entire membrane region. Scale bar: 500 µm.

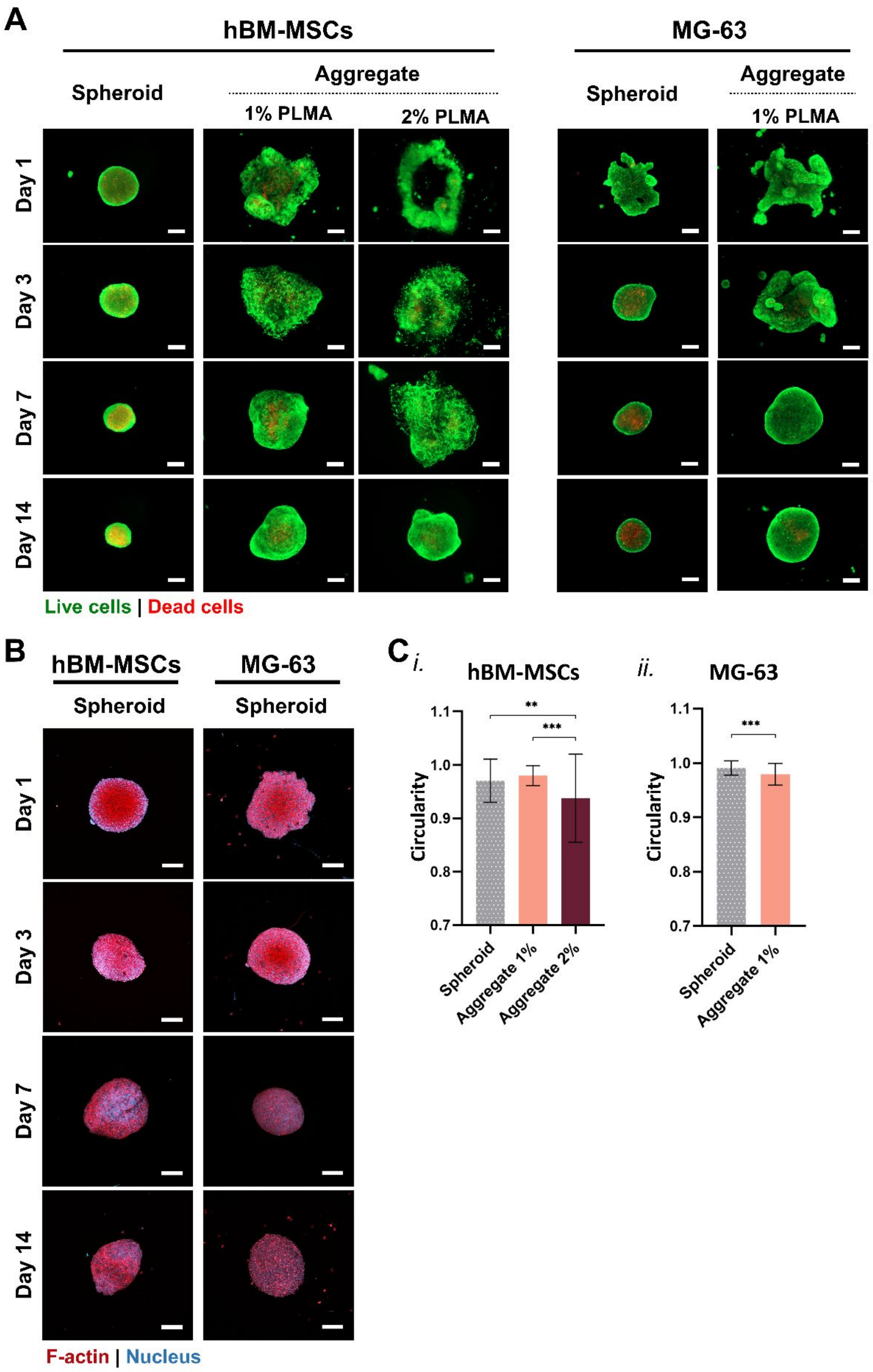

Figure S8. Viability and characterization of the cell aggregates. (A) Widefield fluorescence images of the viability of hBM-MSCs and MG-63 aggregates up to 14 days in culture. Spheroids generated in ultra-low adhesion U-shape plates were used as controls for comparison purposes. Scale bar: 200 µm. (B) Confocal laser microscopy images of the cell spheroids up to 14 days in

culture. Scale bar: 200 µm. (C) Circularity of the hBM-MSCs and MG-63 spheroids and cell aggregates measured at the end of the experiment (14 days of culture). Data are presented as mean ± SD (n ≥ 3). **p < 0.01; ***p < 0.001

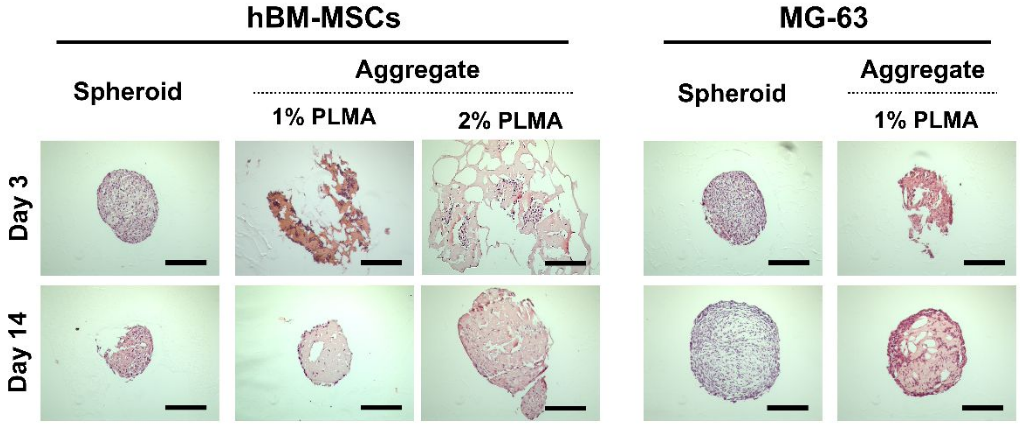

Figure S9. Histological characterization of the 3D microtissues. Hematoxylin and eosin staining of the hBM-MSCs and MG-63 spheroids and aggregates at 3 and 14 days of culture. Scale bar: 200 µm.

References

(1) Gomes, M. C.; Pinho, A. R.; Custódio, C.; Mano, J. F. Self-Assembly of Platelet Lysates Proteins into Microparticles by Unnatural Disulfide Bonds for Bottom-Up Tissue Engineering. *Adv Mater* **2023**, *35* (41), 2304659. https://doi.org/10.1002/adma.202304659.
